# Increased antibody titers but induced T cell AICD and apoptosis response in COVID-19 convalescents by inactivated vaccine booster

**DOI:** 10.1128/spectrum.02435-23

**Published:** 2024-02-06

**Authors:** Jingmin Zhao, Han Zhang, Lina Jiang, Fang Cheng, Wei Li, Zihao Wang, Hongyang Liu, Shaohua Li, Yiyun Jiang, Meiling Li, Yan Li, Shuhong Liu, Min Fang, Xuyu Zhou, Xin Ye, Shousong Zhao, Yuxuan Zheng, Songdong Meng

**Affiliations:** 1 Department of Pathology and Hepatology, The Fifth Medical Center of PLA General Hospital, Beijing, China; 2 CAS Key Laboratory of Pathogen Microbiology and Immunology, Institute of Microbiology, Chinese Academy of Sciences (CAS), Beijing, China; 3 University of Chinese Academy of Sciences, Beijing, China; 4 Department of Infectious Diseases, First Affiliated Hospital of Bengbu Medical College, Bengbu, China; 5 Human Phenome Institute, Fudan University, Shanghai, China; David Geffen School of Medicine at UCLA, Los Angeles, California, USA

**Keywords:** SARS-CoV-2, Omicron, convalescents, vaccine, T cells, cell death

## Abstract

It is urgently needed to evaluate the necessity and benefits of booster vaccination against the coronavirus 2 of the severe acute respiratory syndrome (SARS-CoV-2) Omicron to facilitate clinical decision-making for 2019 coronavirus disease (COVID-19) convalescents. We conducted a multicenter, prospective clinical trial (registration number: ChiCTR2100045810) in the first patients with COVID-19 from 28 January 2020 to 20 February 2020 to assess the long-term durability of neutralizing antibodies against live Omicron BA.5 and further assess the efficiency and safety of CoronaVac in the convalescent group. A total of 96 COVID-19 convalescents were enrolled in this study. Neutralizing antibody titers in convalescents were significantly reduced in 9–10 months. A dose-refreshing vaccination in 28 convalescents with an antibody titer below 96 significantly induced neutralizing antibodies against live Omicron by 4.84-fold. Meanwhile, the abundance of naive T cells increased dramatically, and T_EMRA_ and T_EM_ cells gradually decreased after vaccination. Activation-induced cell death and apoptosis-related genes were significantly elevated after vaccination in all T-cell subtypes. One-dose booster vaccination was effective in inducing a robust antibody response against SARS-CoV-2 Omicron in COVID-19 convalescents with low antibody titers. However, vaccine-mediated T-cell consumption and regeneration patterns may be detrimental to the antiviral response.

**IMPORTANCE:**

The globally dominant coronavirus 2 of the severe acute respiratory syndrome (SARS-CoV-2) Omicron variant raises the possibility of repeat infections among 2019 coronavirus disease (COVID-19) convalescents with low neutralizing antibody titers. The importance of this multicenter study lies in its evaluation of the long-term durability of neutralizing antibodies in COVID-19 convalescents and the efficacy of a booster vaccination against the live Omicron. The findings suggest that a one-dose booster vaccination is effective in inducing a robust antibody response against SARS-CoV-2 Omicron in convalescents with low antibody titers. However, the study also highlights the potential detrimental effects on the antiviral response due to vaccine-mediated T-cell consumption and regeneration patterns. These results are crucial for facilitating clinical decision-making for COVID-19 convalescents and informing public health policies regarding booster vaccinations.

## INTRODUCTION

The 2019 coronavirus disease (COVID-19) pandemic, caused by the new coronavirus 2 of the severe acute respiratory syndrome (SARS-CoV-2), continues to impose worldwide burdens on morbidity and mortality. Within COVID-19 convalescents, both humoral immunity and cellular immunity play essential roles in protection against reinfection (1, 2). The Omicron variant has recently become globally dominant, including in China. Multiple studies have demonstrated that because of mutations in the spike protein, Omicron sublineages BA.4/BA.5 are substantially more resistant to sera from individuals vaccinated with COVID-19 and thus likely lead to breakthrough vaccine infections (3–5).

Vaccination with inactivated whole virus vaccines, protein subunit vaccines, mRNA vaccines, or viral vector-based vaccines in uninfected individuals has been shown to significantly reduce the chances of infection with SARS-CoV-2 or decrease the disease severity after infection (6, 7). However, for COVID-19 convalescents, studies on dynamic antibody changes using live Omicron neutralization tests still need to be completed. While vaccination in COVID-19 convalescents is supported by the Chinese guidelines (8), the WHO and European guidelines do not support such an immune strategy (9, 10). Previous studies of the duration and dynamics of antibody responses showed that a single dose of vaccine induced a strong humoral response against multiple variants of SARS-CoV-2, except for the current dominant Omicron variant (1, 11, 12). In these studies, no investigation of the impact of vaccination on immune status and function was performed to inform vaccine design, as SARS-CoV-2 infection can induce deep and sustained immune dysregulation and alter the function of multiple immune cells, including certain types of T cells, B cells, and innate cells in convalescents (13–15). Therefore, large-scale, equitable access and multicenter investigations are urgently needed to acquire conclusive knowledge of the dynamics of humoral and cellular responses against the Omicron variant in convalescents.

## MATERIALS AND METHODS

### Study design and participants

Ninety-six participants with COVID-19 were enrolled in the study from three designated centers in China. The patient cohort was recruited from 28 January 2020 to 20 February 2020. Among them, 49 participants were admitted from The First Affiliated Hospital of the Bengbu Medical College, 23 from the Fifth Medical Center of PLA General Hospital, and 24 from the Fifth People’s Hospital of Bengbu. The eligibility criteria were that all patients enrolled had a confirmed SARS-CoV-2 infection detected by a nasal swab PCR test. All participants had a definitive history of direct or indirect exposure to the virus through Hubei Province, China. The inclusion criteria for patients with COVID-19 enrolled in this prospective cohort study was confirmed and classified according to the Guidelines for the Diagnosis and Treatment of New Coronavirus Pneumonia (version 5) published by the Chinese National Health Commission (15). Laboratory confirmation of COVID-19 was performed at the Chinese Center for Disease Control and Prevention with RT-PCR detection reagents. Those without complete clinical data of interest were excluded (*n* = 6). All participants (*n* = 90) who met the eligibility criteria were included in the subsequent analysis. Severe patients (Table S1) were diagnosed with at least one of the following criteria: (i) shortness of breath with RR ≥ 30 times/min; (ii) oxygen saturation (resting state) ≤ 93%; or (iii) PaO_2_/FiO_2_ < 300 mmHg. The registration number in ResMan IPD of the cohort is ChiCTR2100045810. Clinical data were monitored, and blood samples were collected at different interval times (admission, the time points required during hospitalization, before discharge, 1 month after discharge, 2–3 months after discharge, 6 months after discharge, 9–10 months after discharge, the day of vaccination, 7 days after vaccination, and 28 days after vaccination). Long COVID-19 is arbitrarily defined as symptoms that extend beyond 12 weeks from initial symptoms (16). Of the 90 convalescent patients with a low neutralized antibody (<1:96), 28 were vaccinated with an inactivated COVID-19 vaccine (CoronaVac, by Sinovac, strain CN02) 9–10 months after discharge.

Epidemiological, clinical, and laboratory data were reviewed from electronic medical records, and data were entered into a standardized collection form. A trained team of physicians reviewed the data. Follow-up consultations were performed by doctors every month after discharge and vaccination. The consultation content was the same as hospital admission, including important signs and symptoms. The final date of follow-up was 30 September 2022. Relative clinical data were collected during follow-up. The primary outcome of this study was to observe the protective function and adverse events of vaccination. The kinetic changes of neutralizing antibodies were monitored in different subpopulations, including severe and non-severe patients. The secondary outcome was to detect the immune responses of B and T cells in COVID-19 patients, thus exploring the mechanisms of vaccination in convalescents.

### Methods

The reagents for cell culture, the antibodies, and constructs, as well as the procedures for performing the live virus neutralization test (VNT) and RBD ELISA, peripheral blood mononuclear cell (PBMC) isolation, flow cytometry analysis, single-cell RNA-seq bioinformatics analysis, and statistical analysis, are described in the supplemental material.

### Live VNT and RBD ELISA

The VNT is assumed to be the reference for assessing the ability of antibodies to block the virus from entering human cells. The results of the SARS-CoV-2 neutralization test show a strong correlation with the protective titer of the test sample. VNT assays were performed by the Sinovac laboratory in China, using live prototypic, Delta, or Omicron variant BA.5. The assay requires a biosafety level 3 laboratory with trained staff and specific equipment. The neutralizing antibody titer was determined with a four-step protocol: Vero epithelial cell line culture, SARS-CoV-2 virus titration, viral growth in cell culture, and microneutralization assay with a subsequent reading of cytopathic effect (CPE).

The outputs were generated as relative titers and were based on CPE induced by the virus on cultured cells. The VNT test was performed on Vero cells from the African green monkey (*Cercopithecus aethiops*). The viruses were titrated in serial log dilutions to obtain a 50% cell culture infective dose (CCID) on 96-well culture plates of Vero cells. The plates were observed for 5 days to evaluate the CPE. The end-point titer was calculated according to the Reed and Muench method.

Serum samples heat-inactivated for 30 min at 56°C were subjected to two-fold serial dilutions from 1:4 to 1:8,192. Serum dilutions were mixed with an equal volume of viral solution containing 100 CCID_50_ of the SARS-CoV-2 virus. The serum-virus mixture was incubated for 2 h at 37°C in a humidified atmosphere with 5% CO_2_. After incubation, 100 µL of each dilution mixture and cells were added to a cell plate in duplicate. The plates were then incubated for 5 days at 37°C in a humidified atmosphere with 5% CO_2_. After 5 days of incubation, the plates were analyzed with an inverted optical microscope. The highest serum dilution that could protect more than 90% of CPE cells was taken as the neutralization titer.

The concentration of RBD-specific antibodies in serum samples was quantitatively measured with a SARS-CoV-2 Spike RBD protein antibody ELISA Kit (RK04144). Briefly, serially diluted serum samples were added to 96-well ELISA plates precoated with recombinant SARS-CoV-2 RBD proteins and incubated for 2 h at room temperature. The plates were washed and incubated with biotin-conjugated RBD protein for 1 h at room temperature. Then, the plates were washed and incubated with diluted streptavidin-HRP working solutions for 0.5 h at room temperature. Finally, the plates were detected with 3,3′,5,5′-tetramethylbenzidine (Invitrogen) and quenched with 2 M H_2_SO_4_. A spectrophotometer measured the optical density (OD) of the sample at 450 nm. The serum concentrations of RBD protein-specific antibodies were determined by a standard curve prepared from seven standard dilutions of control antibody.

## RESULTS

### Serum antibody responses to an inactivated vaccine in COVID-19 convalescents

**Fig 1:**
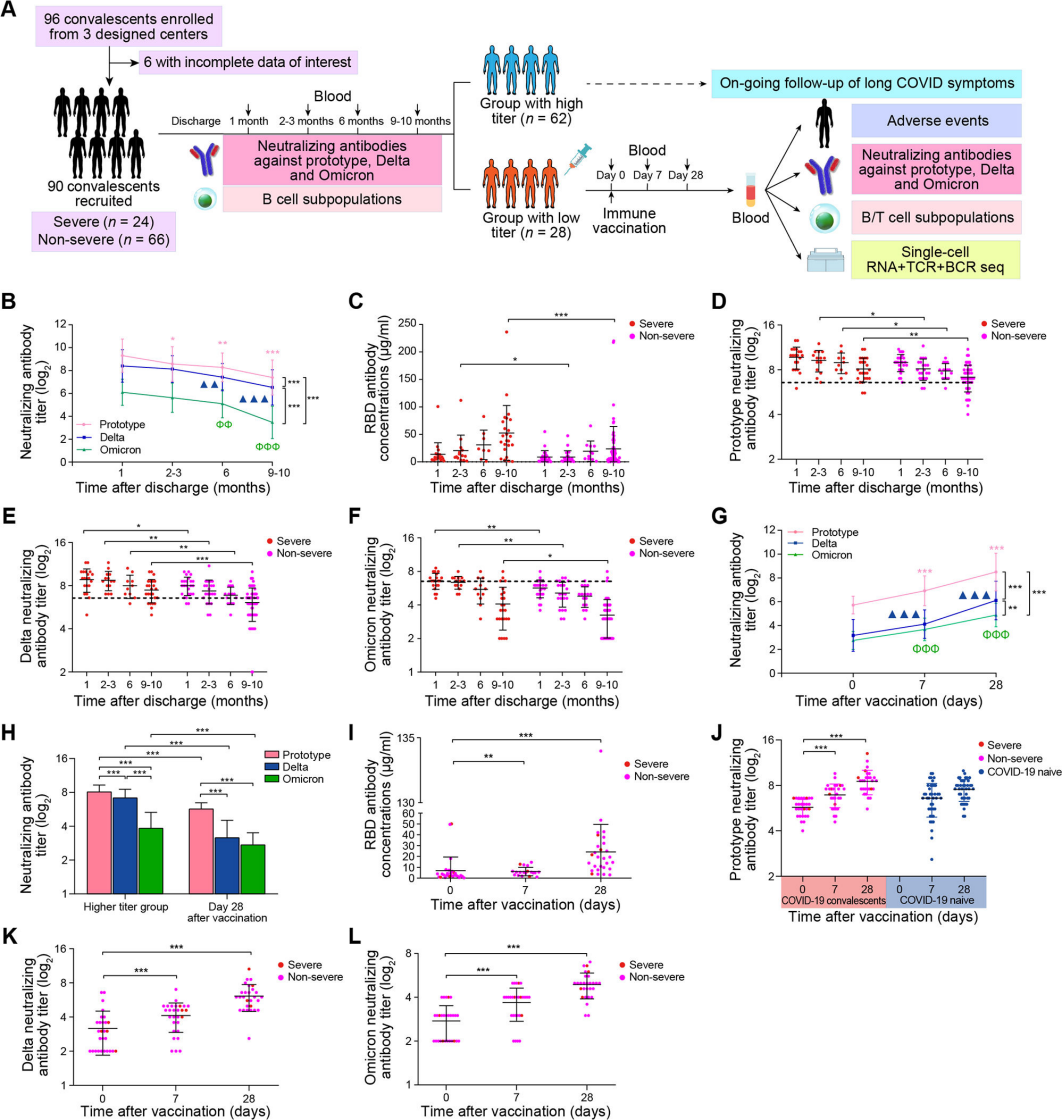
Study design and analysis of the dynamics of antibodies in COVID-19 convalescents. (**A**) Study flow chart, including the process in convalescents and the vaccinated group. A total of *n* = 90 convalescents were included to analyze the dynamics of antibody. Among them, 28 convalescents with a neutralizing antibody titer of less than 1:96 at 9–10 months were chosen for a vaccination boost. (**B**) Line plot showing the neutralization titer against the live SARS-CoV-2 prototypic strain, Delta, or Omicron BA.5 variants in COVID-19 convalescents after discharge. The lines indicate SARS-CoV-2 strains. Data are shown as mean ± SD. Repeated measures ANOVA *P* values (for a given strain, compared to the level at 1 month) and two-tailed Student’s *t*-test *P* values (compared between different strains) were computed (same as panel G). *P < 0.05; **△△ or ϕϕ, *P* < 0.01; and ***△△△ or ϕϕϕ, *P* < 0.001. (**C**) Scatter plot showing the SARS-CoV-2 RBD antibody concentrations examined by ELISA in COVID-19 convalescents after discharge. The colored dots indicate severe or non-severe convalescents. Data are shown as mean ± SD. Repeated measures ANOVA *P* values (for a given group, compared with the level at 1 month) and two-tailed Student’s *t*-test *P* values (compared between severe and non-severe groups) were computed (same as panels D–F). *P < 0.05 and ***P < 0.001. (**D–F**) Scatter plots showing the neutralization titer against live prototypic strain (**D**), Delta (**E**), or Omicron BA.5 variants (**F**) in COVID-19 convalescents after discharge. The colored dots indicate severe or non-severe convalescents. The dotted lines indicate the value of log_2_ 96 of neutralizing antibody titer. Data are shown as mean ± SD. *P < 0.05; **P < 0.01; and ***P < 0.001. (**G**) Line plot showing the neutralization titer against live prototypic strain, Delta, or Omicron BA.5 variants in COVID-19 convalescents after vaccination. The lines indicate SARS-CoV-2 strains. Data are shown as mean ± SD. **P < 0.01 and ***△△△ or ϕϕϕ, *P* < 0.001. (**H**) Bar plot showing the neutralization titer against live prototypic strain, Delta, or Omicron BA.5 variants in COVID-19 convalescents after discharge or vaccination. Data are shown as mean ± SD. The ANOVA *P* values (for a given group, compared among different strains) and two-tailed Student’s *t*-test *P* values (compared between groups) are indicated. ***P < 0.001. (**I**) Scatter plot showing the SARS-CoV-2 RBD antibody concentrations examined by ELISA in COVID-19 convalescents after vaccination. The colored dots indicate severe or non-severe convalescents. Data are shown as mean ± SD. Repeated measures of the ANOVA *P* values are indicated. **P < 0.01 and ***P < 0.001. (**J–L**) Scatter plots showing the neutralization titer against live prototypic strain (**J**), Delta (**K**), or Omicron BA.5 variants (**L**) in COVID-19 convalescents or COVID-19 naive individuals after vaccination. Data are shown as mean ± SD. Repeated measures ANOVA *P* values (for a given group, compared with the level at day 0) and two-tailed Student’s *t*-test *P* values (compared between convalescents and COVID-19 naive individuals; only for panel J) are computed. ***P < 0.001.

We first investigated the dynamics of antibody titers in COVID-19 convalescents up to 1 year after discharge. Ninety individuals were recruited, and all consented to outpatient follow-up and collection of serial blood samples (Fig. 1A; Table 1; Table S1). Among 90 patients, 24 were diagnosed with severe COVID-19 (Table S2). Our results of neutralizing antibody titer against prototypic strain, Delta, and Omicron variants in convalescents revealed a dynamic reduction by 3.44-fold (300.18 vs 1,033.45, 9–10 months vs 1 month, *P* < 0.001), 3.25-fold (166.58 vs 541.82, 9–10 months vs 1 month, *P* < 0.001), and 4.22-fold (22.00 vs 92.89, 9–10 months vs 1 month, *P* < 0.001), respectively (Fig. 1B). Unlike the RBD antibody titer (Fig. 1C), the neutralizing antibody titer of prototypic strain (Fig. 1D), Delta (Fig. 1E), and Omicron (Fig. 1F) gradually decreased in both groups, with the severe patients possessing higher antibody titer than non-severe patients. A previous study showed that as long as the serum-neutralizing antibody level reaches or is higher than 20.2% of the average serum-neutralizing antibody level in convalescents, it has more than a 50% protective effect (17). Among the neutralizing antibody titers we tested, 1:96 was in line with this range threshold, so we defined convalescents with neutralizing antibody titers lower than 1:96 as a low-protective group.

**TABLE 1:** Baseline characteristics and laboratory findings of patients^*a*^

Characteristics	Total *N* = 90	Low level of 1:96 *N* = 28	High level of 1:96 *N* = 62	*P*
Age (year)	54 (46–61)	54 (48–58)	54 (45–62)	0.568
Sex (male)	42 (46.67)	16 (57.14)	26 (41.94)	0.181
Severe	24 (26.67)	4 (14.29)	20 (32.26)	0.074
Exposure				
Hubei province exposure	8 (8.89)	0 (–)	8 (12.90)	0.112
Close contact with COVID-19 patient	35 (38.89)	13 (46.43)	22 (35.48)	0.324
All of the above	4 (4.44)	0 (–)	4 (6.45)	0.306
No exposure	51 (56.66)	15 (53.57)	36 (58.06)	0.691
Coexisting medical conditions				
Diabetes	7 (7.78)	1 (3.57)	6 (9.68)	0.428
Hypertension	18 (20.00)	6 (21.43)	12 (19.35)	0.820
Cardiovascular disease	6 (6.67)	1 (3.57)	5 (8.06)	0.661
Endocrine system disease	2 (2.22)	0 (–)	2 (3.23)	1.000
Respiratory system disease	8 (8.89)	3 (10.71)	5 (8.06)	0.993
Signs and symptoms				
Fever	64 (71.11)	21 (75.00)	43 (69.35)	0.584
Cough	50 (55.56)	13 (46.43)	37 (59.68)	0.242
Myalgia or fatigue	34 (37.78)	10 (35.71)	24 (38.71)	0.786
Sputum production	26 (28.89)	7 (25.00)	19 (30.65)	0.584
Headache	9 (10.00)	3 (10.71)	6 (9.68)	1.000
Diarrhea	6 (6.67)	3 (10.71)	3 (4.84)	0.370
Dyspnea	14 (15.56)	4 (14.29)	10 (16.13)	1.000
Sore throat	6 (6.67)	2 (7.14)	4 (6.45)	1.000
Rhinorrhea	3 (3.33)	1 (3.57)	2 (3.23)	1.000
Nausea and vomiting	6 (6.67)	0 (–)	6 (9.68)	0.171
Chest pain and stuffiness	8 (8.89)	2 (7.14)	6 (9.68)	1.000
Treatment				
PEG-interferon α2b	70 (77.78)	23 (82.14)	47 (75.81)	0.503
Lopinavir/ritonavir	48 (53.33)	14 (50.00)	34 (54.84)	0.670
Antibiotic treatment	35 (38.89)	10 (35.71)	25 (40.32)	0.678
Use of corticosteroid	5 (5.56)	0 (–)	5 (8.06)	0.319
Intravenous immunoglobulin therapy	6 (6.67)	1 (3.57)	5 (8.06)	0.661
Chinese traditional medicine	56 (62.22)	18 (64.29)	38(61.29)	0.786
Long COVID-19	11 (12.22)	2 (7.14)	9 (14.52)	0.522
Biochemistry				
TP 60–83 (g/L)	68.79 (64.35–71.85)	68.53 (63.62–72.26)	68.79 (64.70–71.50)	0.616
Albumin 35–55 (g/L)	38.00 (34.50–40.40)	38.90 (36.55–40.70)	36.85 (34.30–40.20)	0.126
Globulin 20–40 (g/L)	30.46 (26.80–33.83)	29.82 (25.03–35.16)	30.70 (26.98–33.71)	0.714
A/G 1.2–2.4	1.26 (1.08–1.49)	1.25 (1.11–1.64)	1.26 (1.07–1.40)	0.333
DBiL 0–6.8 (μmol/L)	3.15 (1.90–5.60)	2.90 (1.80–5.60)	3.20 (2.12–5.60)	0.672
TBiL 3.4–20.5 (μmol/L)	9.72 (6.90–13.50)	9.81 (7.51–14.20)	9.71 (6.44–13.50)	0.454
D/T	0.37 (0.25–0.48)	0.31 (0.22–0.44)	0.39 (0.25–0.50)	0.173
ALT 5–40 (U/L)	23.85 (15.00–38.30)	32.25 (14.65–47.70)	21.80 (15.00–36.20)	0.205
AST 5–40 (U/L)	31.50 (22.00–41.90)	28.90 (20.90–47.65)	31.90 (22.60–40.60)	0.760
S/T	0.94 (0.70–1.45)	1.06 (0.81–1.51)	0.91 (0.64–1.45)	0.086
ALP 40–150 (U/L)	54.00 (40.00–69.00)	59.00 (45.00–77.50)	52.00 (39.00–63.00)	0.138
GGT 11–50 (U/L)	26.00 (18.00–45.00)	22.50 (15.00–33.00)	29.50 (19.00–51.00)	0.086
Cholinesterase 5,000–12,000 (U/L)	6,532 (5,872–7,548)	6,678 (5,679–7,906)	6,532(5,942–7,325)	0.604
Urea 2.9–8.2 (mmol/L)	4.05 (3.30–4.69)	4.18 (3.70–4.63)	3.75 (3.10–4.86)	0.178
Creatinine 62–115 (μmol/L)	58.70 (50.00–70.00)	62.00 (54.00–69.00)	57.30 (48.40–70.00)	0.494
UA 208–428 (μmol/L)	219.50 (179.00–264.00)	231 (203–263)	218 (169–264)	0.280
Glucose 3.9–6.1 (mmol/L)	5.95 (5.21–7.20)	5.60 (5.05–6.12)	6.10 (5.35–7.80)	0.007
TC 2.8–5.2 (mmol/L)	3.70 (3.35–4.15)	3.71 (3.35–4.12)	3.69 (3.33–4.23)	0.963
Triglyceride 0.56–1.7 (mmol/L)	1.13 (0.88–1.61)	1.13 (0.88–1.69)	1.10 (0.85–1.52)	0.834
HDL-c 1.29–1.55 (mmol/L)	1.06 (0.85–1.21)	1.06 (0.84–1.23)	1.05 (0.85–1.21)	0.759
LDL-c 2.1–3.1 (mmol/L)	2.14 (1.80–2.55)	2.21 (1.90–2.39)	2.07 (1.79–2.67)	0.638
Apo-A1 1.05–2.05 (g/L)	0.92 (0.82–1.07)	1.00 (0.85–1.07)	0.90 (0.80–1.06)	0.168
Apo-B 0.55–1.30 (g/L)	0.79 (0.65–0.95)	0.79 (0.64–1.01)	0.78 (0.65–0.93)	0.571
ADA 0–20 (U/L)	21.15 (16.75–23.95)	20.70 (16.40–22.95)	21.30 (16.75–24.25)	0.707
Calcium 2.03–2.54 (mmol/L)	2.16 (2.10–2.24)	2.18 (2.14–2.27)	2.16 (2.09–2.22)	0.112
Phosphorus 0.97–1.45 (mmol/L)	0.97 (0.87–1.10)	1.00 (0.89–1.15)	0.96 (0.86–1.09)	0.507
Magnesium < 1.25 (mmol/L)	0.92 (0.84–1.02)	0.95 (0.88–1.02)	0.92 (0.84–1.01)	0.393
CK 18.0–198.0 (U/L)	77 (49–165)	71 (52–161)	83 (44–175)	0.935
CKMB < 0.6 ng/mL	7.55 (5.00–11.00)	8.70 (5.30–11.30)	7.50 (4.20–11.00)	0.542
NT-proBNP 0–125 (pg/mL)	95 (42–224)	119 (45–274)	66 (33–176)	0.527
LDH 109–245 (U/L)	278 (227–340)	245 (201–292)	301 (247–366)	0.009
CRP 0.068–8.2 (mg/L)	27.72 (12.08–77.63)	19.90 (5.25–31.47)	33.94 (16.50–82.10)	0.027
ESR 0–15 (mm/60 min)	46.85 (27.00–76.80)	41.45 (17.70–71.60)	46.85 (33.60–81.90)	0.368
Blood routine				
WBC 3.97–9.15 (10^9^/L)	5.28 (4.26–6.99)	5.26 (4.27–7.69)	5.29 (4.14–6.76)	0.506
NEUT 2–7 (10^9^/L)	3.62 (2.49–4.47)	3.54 (2.12–4.81)	3.66 (2.61–4.39)	0.910
LYMPH 0.8–4.0 (10^9^/L)	1.24 (0.88–1.69)	1.54 (0.89–2.05)	1.16 (0.87–1.58)	0.105
MONO 0.12–1.0 (10^9^/L)	0.37 (0.25–0.57)	0.40 (0.26–0.57)	0.35 (0.23–0.56)	0.566
EO 0.02–0.5 (10^9^/L)	0.02 (0.01–0.08)	0.02 (0.01–0.10)	0.02 (0.01–0.07)	0.358
BASO 0–1 (10^9^ /L)	0.01 (0.01–0.02)	0.02 (0.01–0.03)	0.01 (0.01–0.02)	0.025
RBC 4.09–5.74 (10^12^/L)	4.16 (3.85–4.51)	4.45 (3.85–4.81)	4.11 (3.85–4.38)	0.069
Hemoglobin 131–172 (g/L)	131 (118–140)	137 (118–148)	129 (118–136)	0.075
PCV 38–50.8 (%)	39.00 (35.65–42.15)	41.00 (35.80–44.30)	38.50 (35.30–41.20)	0.054
MCV 83.9–99.1 (fL)	93.15 (90.25–96.25)	93.00 (90.00–96.80)	93.30 (90.30–96.20)	0.867
MCHC 27.8–33.8 (pg)	31.00 (29.90–31.95)	30.80 (29.80–31.70)	31.00 (29.90–32.00)	0.609
RDW 35.0–56.0 (fL)	42.60 (40.00–44.70)	41.70 (39.80–44.70)	42.80 (40.00–44.70)	0.556
PLT 85–303 (10^9^/L)	160 (129–204)	153 (138–190)	160 (119–210)	0.935
PCT 0.06–0.40 (%)	0.02 (0.02–0.15)	0.02 (0.02–0.15)	0.02 (0.02–0.16)	0.819
MPV 7.54–11.24 (fL)	9.30 (8.85–10.35)	9.30 (9.00–9.90)	9.30 (8.80–10.50)	0.914
PDW 9.0–18.0 (%)	16.10 (15.70–16.30)	16.10 (15.80–16.30)	16.10 (15.70–16.30)	0.803
Coagulation routine				
PT 11–13 (s)	12.05 (11.40–12.80)	12.10 (11.40–13.00)	11.90 (11.40–12.80)	0.883
INR 0.8–1.2	1.05 (1.00–1.11)	1.05 (1.00–1.10)	1.05 (1.00–1.13)	0.873
D-dimer < 0.55 (mg/L)	0.56 (0.31–0.86)	0.44 (0.27–0.52)	0.69 (0.40–0.96)	0.010

^*a*^ ADA, adenosine deaminase; ALT, alanine aminotransferase; AST, aspartate aminotransferase; ALP, alkaline phosphatase; Apo B, apolipoprotein B; Apo A1, apolipoprotein A1; BNP, brain natriuretic peptide; CRP, C-reactive protein; CK, creatine kinase; DBiL, direct bilirubin; ESR, erythrocyte sedimentation rate; EO, absolute eosinophil; GGT, γ-glutamyl transferase; HDL-c, high density lipoprotein cholesterol; INR, international normalized ratio; LDL-c, low density lipoprotein cholesterol; LDH, lactate dehydrogenase; LYMPH, absolute lymphocyte value; MCV, mean corpuscular volume; MCHC, mean corpuscular hemoglobin concentration; MPV, mean platelet volume; MONO, absolute monocyte; NEUT, absolute neutrophil count; PCV, packed cell volume; PLT, platelet count; PCT, platelet hematocrit; PDW, platelet distribution width; PT, prothrombin time; TP, total protein; TBiL, total bilirubin; TC, total cholesterol; RBC, red blood cell; RDW, red blood cell distribution width; UA, uric acid; WBC, white blood cell. Data and samples of severe patients were collected on the day when they were diagnosed to be severe. Data and samples of non-severe patients were collected at admission.

We found that compared to patients with a low neutralizing antibody titer (<1:96; *n* = 28), those with a high titer (≥1:96; *n* = 62) had a higher frequency of long COVID-19 symptoms (14.52% vs 7.14%; Table S2). Approximately 31.11% (28/90) of convalescents may have reduced virus protection due to a neutralizing antibody titer below 1:96 (17). Of these, 24 convalescents belonged to the non-severe group and only 4 were severe patients. Therefore, vaccination with an inactivated COVID-19 vaccine was carried out to increase protection in these convalescents.

Mild adverse events occurred in 10.71% (3/28) of immunized convalescents (Table S3). The following adverse events were observed: transient headache (1/28, 3.57%) and elevation of transient body temperature (2/28, 7.14%; Table S3). Compared to day 0 (before vaccination), convalescents had a 13.03-fold (774.29 vs 59.43, *P* < 0.001), 9.27-fold (147.64 vs 15.93, *P* < 0.001), and 4.84-fold (37.29 vs 7.71, *P* < 0.001) increase in neutralization activity against the prototypic strain, Delta, and Omicron, respectively, 28 days after immunization. The Omicron was more evasive to neutralization compared to the prototypic strain (20.76-fold) and Delta (3.96-fold) at 28 days after immunization (Fig. 1G). After vaccination, the neutralization antibody titer increased and reached a higher level but was still lower than in 62 convalescents whose neutralization titer was greater than 1:96 at 9–10 months (Fig. 1H). The levels of RBD-specific antibody against the prototypic strain (Fig. 1I) and the neutralizing antibody titer against the prototypic strain (Fig. 1J), Delta (Fig. 1K), and Omicron BA.5 (Fig. 1L) all continued to increase. They peaked 28 days after immunization in both severe and non-severe groups.

### Dynamic changes in B- and T-cell subsets in COVID-19 convalescents after booster vaccination

**Fig 2:**
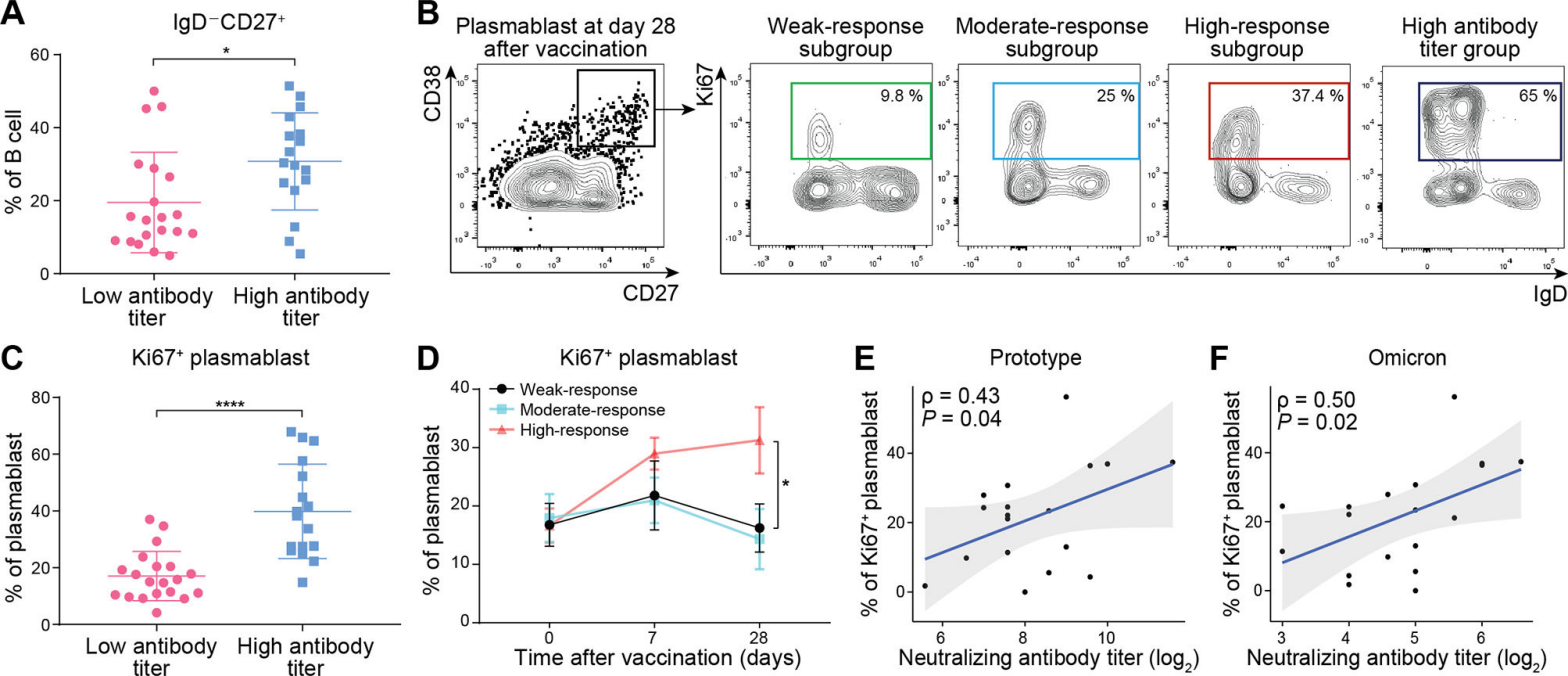
Analysis of B-cell subsets in COVID-19 convalescents. (**A**) Scatter plot showing the frequency of switched memory B cells between the low (*n* = 20) and high (*n* = 17) antibody titer groups. Data are shown as mean ± SD. A two-tailed Student’s *t*-test *P* value is indicated. *P < 0.05. (**B**) Representative data for the gating strategy of Ki67^+^ plasmablasts collected 28 days after vaccination. (**C**) Scatter plot showing the frequency of Ki67^+^ plasmablasts between the low and high antibody titer groups. Data are shown as mean ± SD. A two-tailed Student’s *t*-test *P* value is indicated. ****P < 0.0001. (**D**) Line plot showing the frequency of Ki67^+^ plasmablasts in the three vaccine subgroups of weak (*n* = 7), moderate (*n* = 6), and high response (*n* = 7) to vaccination. Data are shown as mean ± SEM. Statistical significance was calculated between weak and high response subgroups, and a one-way ANOVA *P* value is indicated. *P < 0.05. (**E and F**) Scatter plots showing Pearson’s correlation analysis results on one side between the abundance of Ki67^+^ plasmablasts and prototypic (**E**) or Omicron (**F**) neutralizing antibody titer 28 days after vaccination. The correlation coefficient and the *P* value of the correlation test are indicated.

To clarify the potential correlations between antibody titers and the status of different B-lymphocyte subpopulations, we next analyzed the dynamic changes of major B-cell subsets in different groups. Our results showed that the abundance of switched memory B cells (IgD^-^CD27^+^) was significantly higher in the high antibody titer group than in the low antibody titer group. In contrast, the other B-cell populations showed similar abundance in these two groups (Fig. 2A; Fig. S1A). Next, the low antibody titer group immunized with one dose of COVID-19 inactivated vaccine was divided into three vaccine response subgroups (weak-, moderate-, and high-response subgroups) according to the fold changes in the neutralizing antibody titer before and after vaccination. The abundance of four major B-cell subsets and plasmablasts in these vaccine response subgroups at 0, 7, and 28 days after immunization did not show significant differences (Fig. S1B and S2A). Interestingly, although four main B-cell subsets exhibited similar levels of Ki-67 expression in these vaccine response subgroups (Fig. 2B; Fig. S2B), the abundance of Ki-67^+^ plasmablasts in the high antibody titer group was much higher than in the low antibody titer group (Fig. 2C and D). A clear correlation was observed between the abundance of Ki-67^+^ plasmablasts and the neutralizing antibody titer against prototype and Omicron 28 days after immunization (Fig. 2E and F), indicating that Ki-67 expression in plasmablasts could reflect the antibody titer.

**Fig 3:**
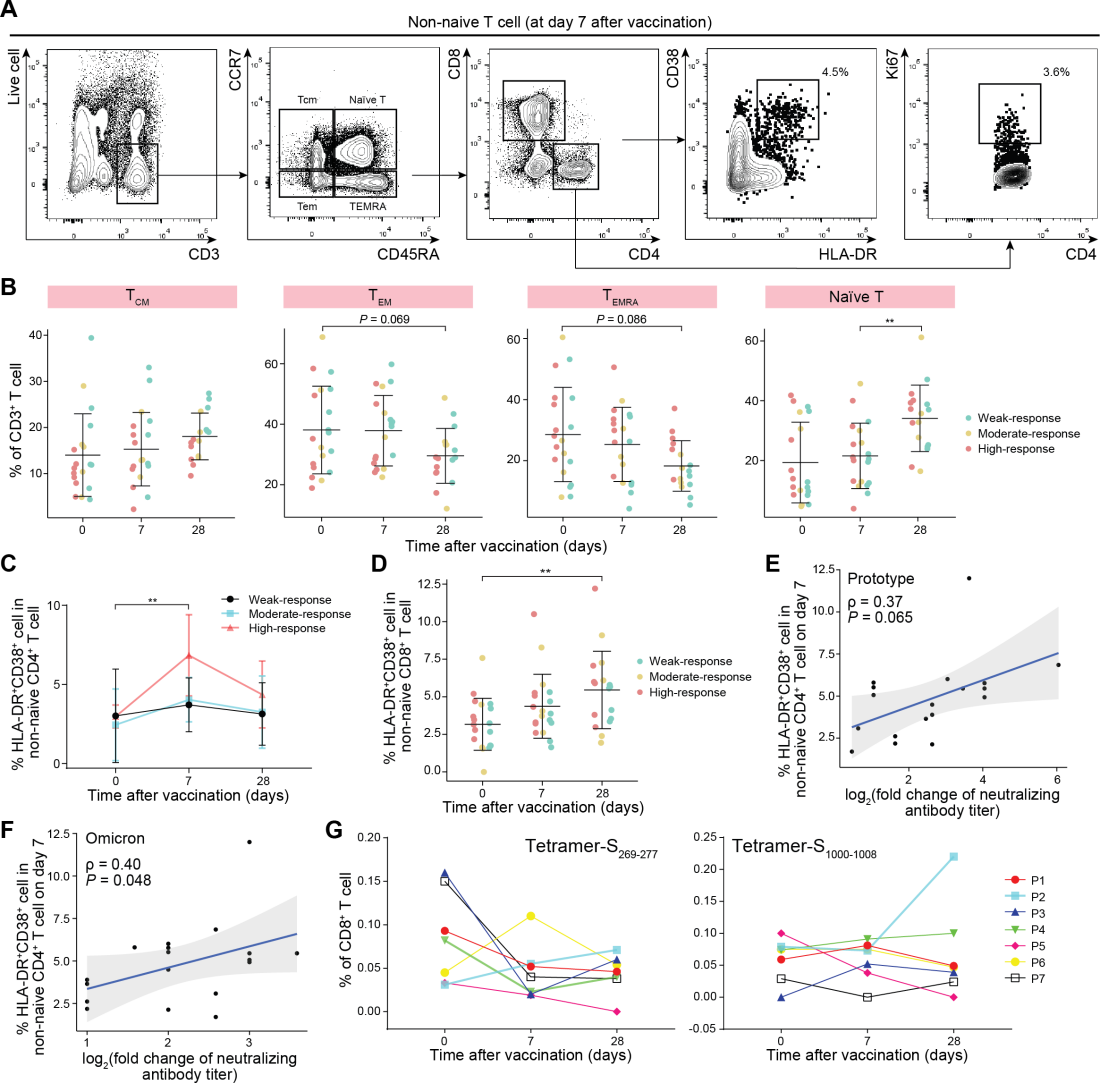
Dynamic changes in the subsets of T cells in vaccinated COVID-19 convalescents. (**A**) Gating strategies of different T-cell subsets collected 7 days after vaccination. (**B**) Scatter plots showing the frequency of different T-cell subsets in three vaccine subgroups of weak, moderate, and high responses to vaccination. The dots indicate the subgroups of vaccine response. Data are shown as mean ± SD. The two-tailed Student’s *t*-test *P* values are indicated. **P < 0.01. (**C**) Line graph showing the frequency of non-naive HLA-DR^+^CD38^+^ CD4^+^ T cells in three subgroups of vaccine responses. Data are shown as mean ± SD. The statistical significance was calculated between days 0 and 7 in the high-response subgroup, and the one-way ANOVA *P* value is indicated. **P < 0.01. (**D**) Scatter plot showing the frequency of non-naive HLA-DR^+^CD38^+^ CD8^+^ T cells in three subgroups of vaccine responses. Data are shown as mean ± SD. A two-tailed Student’s *t*-test *P* value is indicated. **P < 0.01. (**E and F**) Scatter plots showing the results of Pearson’s correlation analysis on one side between the abundance of non-naive HLA-DR^+^CD38^+^ CD4^+^ T cells and the prototypic (**E**) or Omicron (**F**) changes in neutralizing antibody fold on day 7 after vaccination. The correlation coefficient and the *P* value of the correlation test are indicated. (**G**) Line plots showing the SARS-CoV-2-specific CD8^+^ T-cell response detected by tetramer staining before and after vaccination. The lines indicate vaccinated convalescents.

Since natural infection of SARS-CoV-2 could prime both humoral and cellular immune responses simultaneously, we further analyzed the dynamic changes of different subpopulations of T cells in convalescents after one-dose booster vaccination. Surprisingly, the abundance of naive T cells (CD45RA^+^CCR7^+^) increased dramatically 28 days after vaccination (*P* < 0.01). In contrast, the abundance of effector memory cells re-expressing CD45RA (T_EMRA_) (CD45RA^+^CCR7^-^) and effector memory (T_EM_) (CD45RA^-^CCR7^-^) T cells gradually decreased over time and reached the lowest point at 28 days (Fig. 3A and B). The changing trend was similar across three subgroups of vaccine response (Fig. S3A through D).

The abundance of non-naive CD4^+^HLA-DR^+^CD38^+^ T cells increased 7 days after vaccination and then decreased slightly over time, with the vaccine high-response subgroup exhibiting the most apparent activation of T cells. The abundance of non-naive CD8^+^HLA-DR^+^CD38^+^ T cells increased continuously throughout the observation (Fig. 3C and D). Pearson’s correlation analysis showed a positive correlation between the abundance of non-naive CD4^+^HLA-DR^+^CD38^+^ T cells and the fold changes in the neutralizing antibody titer against the prototype and Omicron BA.5 at day 7 but not day 28 (Fig. 3E and F), indicating that the activation of non-naive CD4^+^ T cells may be involved in the vaccine-induced antibody response. The S_269-277_ and S_1000-1008_ peptides derived from the S protein have been identified as HLA-A2-restricted immunodominant epitopes conserved in the variants of SARS-CoV-2 (18). However, no visible SARS-CoV-2-specific cytotoxic lymphocyte response to vaccination was detected by tetramer staining in most HLA-A2-positive convalescents (Fig. 3G).

### Single-cell RNA sequencing reveals dynamic changes in immune cells and distinct response patterns in convalescents after vaccination

**Fig 4:**
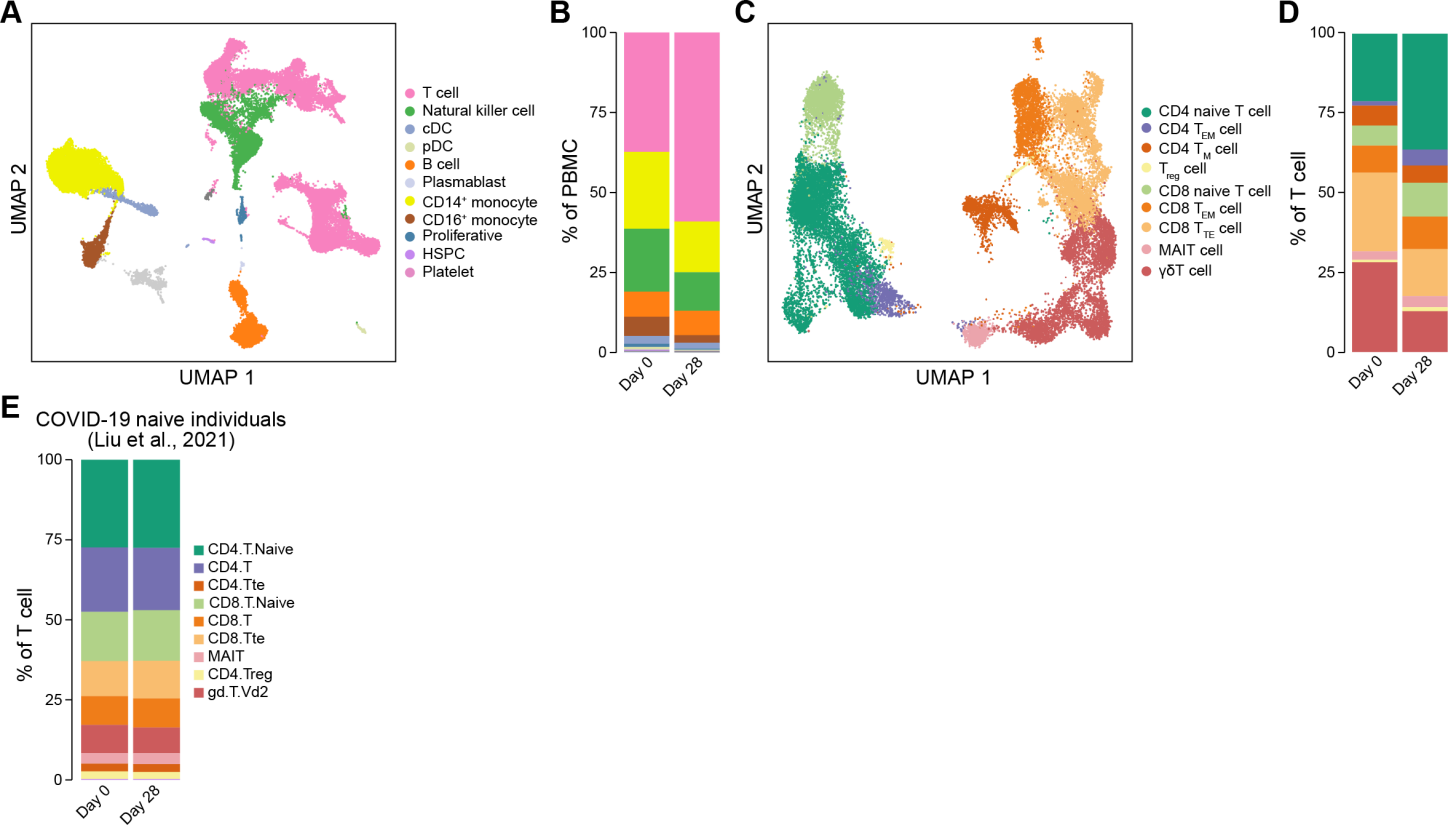
Single-cell RNA-seq analysis of transcriptomic characteristics of vaccinated COVID-19 convalescents. (**A**) UMAP plot showing the main cell type information. The colored dots indicate cell types. (**B**) Stack bar plot showing the proportion of different cell types among all PBMCs before and after vaccination. (**C**) UMAP plot showing the information about the T-cell subtype. The dot color indicates T-cell subtypes. (**D**) Stack bar plot showing the proportion of distinct T-cell subtypes among all T cells before and after vaccination. (**E**) Stack bar plot showing the proportion of distinct T-cell subtypes among all T cells in COVID-19 naive individuals before and after vaccination. Cell information was obtained from the previous study.

To further investigate the global molecular characteristics of immune cells after vaccination, we generated a single-cell combined transcriptome [single-cell RNA-seq (scRNA-seq), single T cell receptor-seq (scTCR-seq), and single B cell receptor-seq (scBCR-seq)] for three convalescent patients who experienced the most obvious increases in naive T cells before (day 0) and after vaccination (day 28) using 10× Genomics platform (Fig. 4A; Fig. S4A through D). We found a decrease in CD14^+^ monocytes and natural killer cells and an increase in T cells (Fig. 4B; Fig. S5A and B), which was not observed in COVID-19 naive individuals after vaccination (19) (Fig. S5C).

We observed the increased abundance of CD4^+^ and CD8^+^ naive T cells, CD4^+^ and CD8^+^ T_EM_ cells, and mucosal-associated invariant T cells after vaccination. However, we noticed decreased CD8^+^ terminal effector T (T_TE_) cells and γδ T cells (Fig. 4C and D; Fig. S6A through D). Furthermore, this was not observed in COVID-19 naive individuals who received multiple boosters (Fig. 4E) (19), suggesting that this T cell response pattern may be specific to COVID-19 convalescents.

**Fig 5:**
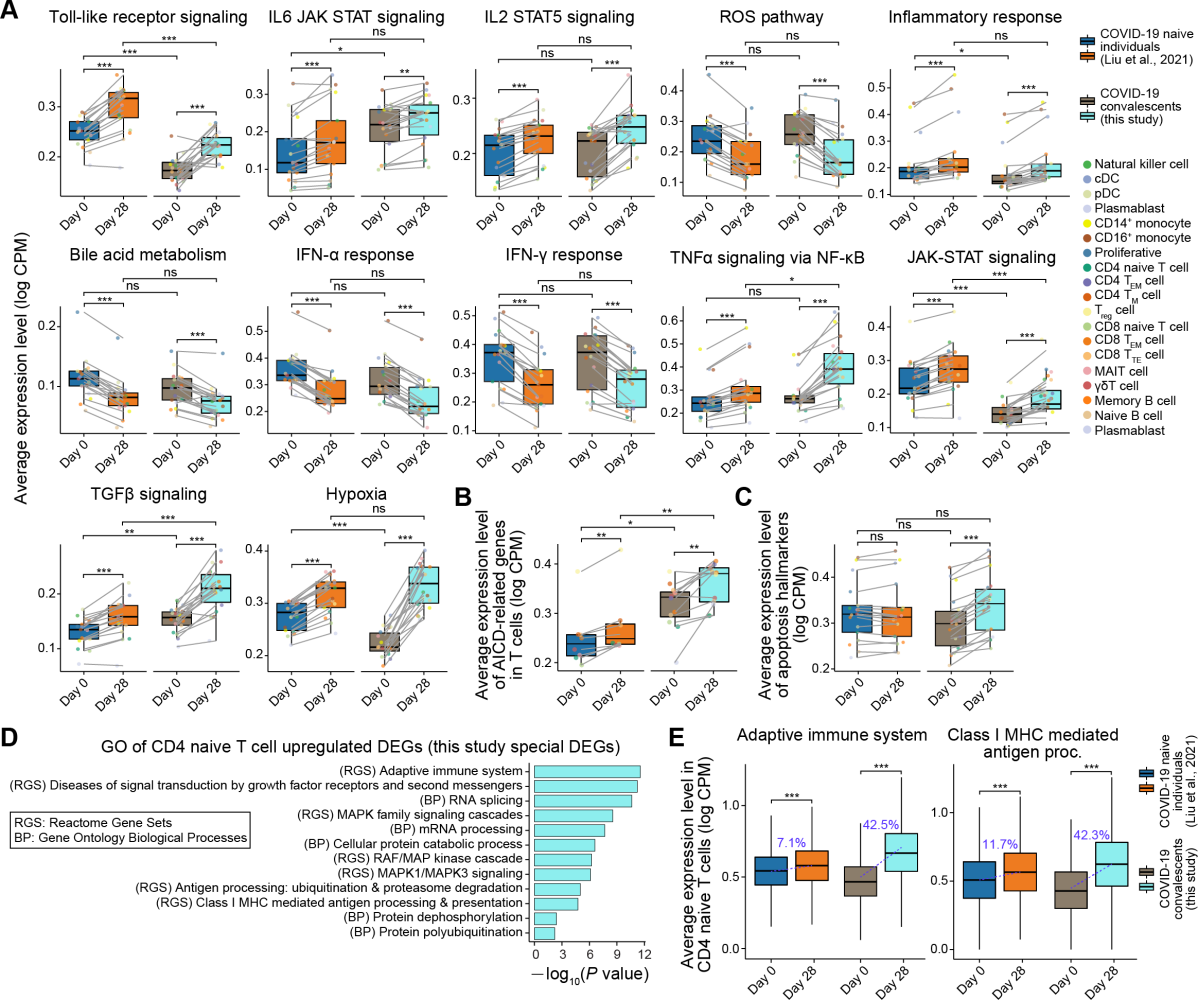
Transcriptomic modifications in COVID-19 convalescents before and after vaccination. (**A–C**) Boxplots showing the average expression level of representative gene sets in each cell type from COVID-19 naive individuals and COVID-19 convalescents. Boxplot color indicates sample information (before or after vaccination and COVID-19 naive individuals or convalescents), and dot color indicates cell type. The lines link the same cell type. Paired Wilcoxon test *P* values are indicated. *P < 0.05; ***P < 0.001; and ns, not significant. (**D**) Bar plot showing the Gene Ontology (GO) terms of the upregulated DEGs in CD4^+^ naive T cell after vaccination. These DEGs were the special DEGs of COVID-19 convalescents in contrast to COVID-19 naive individuals. (**E**) Boxplots showing the average expression level of representative gene sets in CD4^+^ naive T cells of COVID-19 naive individuals and COVID-19 convalescents. Boxplot color indicates the sample information. The blue line and the text indicate the increase in the average expression level. Paired Wilcoxon test *P* values are indicated. ***P < 0.001.

The transcriptomic changes due to vaccination in convalescents showed distinct patterns from those of COVID-19 naive individuals (Fig. S8). The expression level of TNFα signaling via NF-κB signaling pathway-related genes was comparable in COVID-19 naive individuals and COVID-19 convalescents before vaccination, but that level was significantly higher in convalescents than in COVID-19 naive individuals after vaccination (Fig. 5A). The expression levels of activation-induced cell death (AICD)-related genes possibly mediated by TNF pathway (20, 21) were significantly elevated by vaccination in all T cell subtypes of both COVID-19 naive individuals and COVID-19 convalescents, but their expression levels in convalescents were much higher than those in COVID-19 naive individuals (*P* = 4.1 × 10^−3^) (Fig. 5B). Furthermore, significantly increased expression of genes related to apoptosis was observed in all subtypes of T cells in convalescents but not in COVID-19 naive individuals after vaccination (Fig. 5C). In addition, the expression level of genes involved in the signaling pathways of the JAK-STAT and Toll-like receptor in convalescents was lower than in COVID-19 naive individuals before and after vaccination (Fig. 5A), indicating that even after vaccination, part of the dysfunctional immune regulator pathways disrupted by SARS-CoV-2 infection was not fully restored.

**Fig 6:**
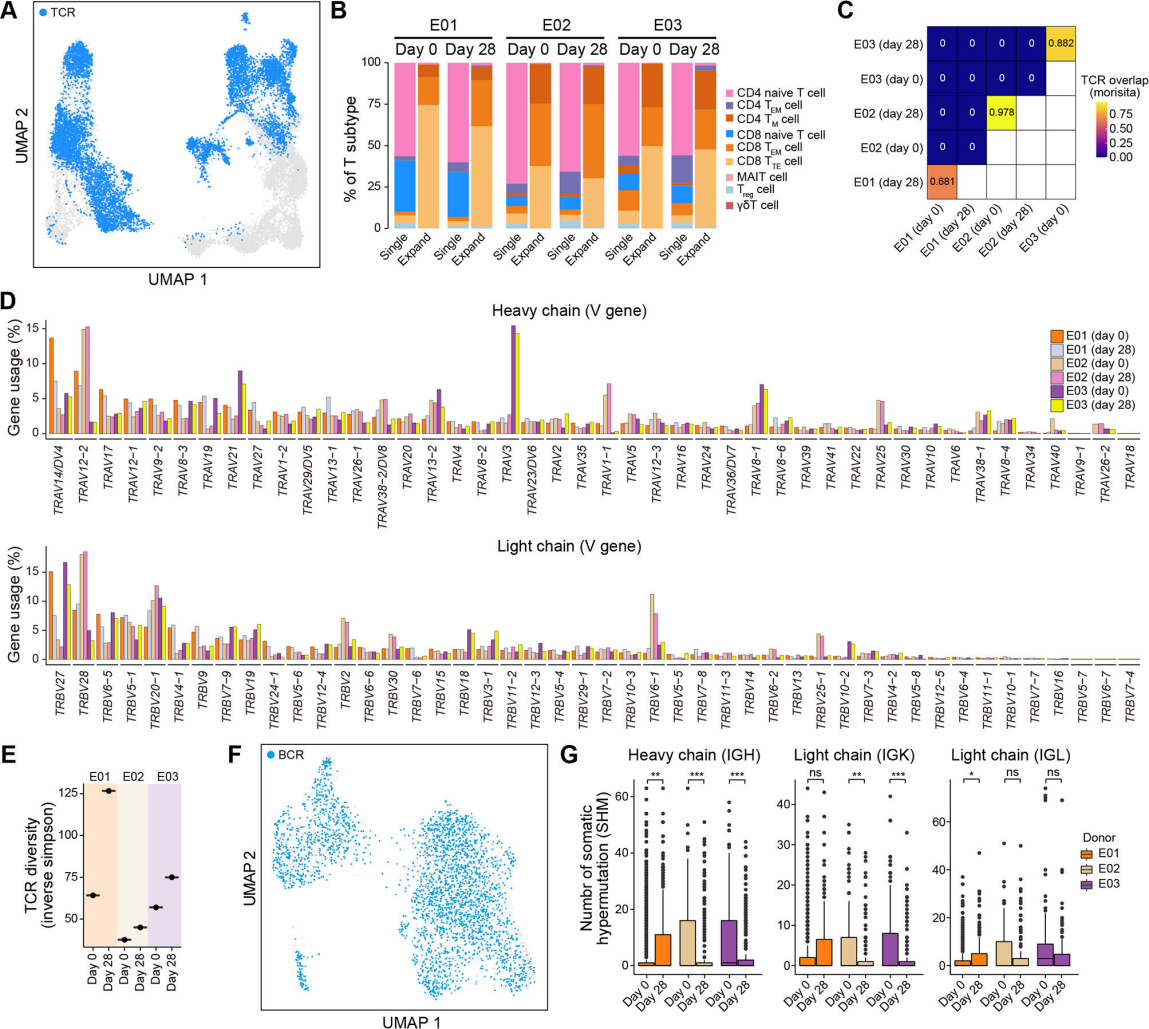
The scTCR-seq and scBCR-seq analysis. (**A**) UMAP plot showing the distribution of high-quality TCRs among all T cells from vaccinated COVID-19 convalescents. (**B**) Stack bar plot showing the proportion of distinct T-cell subtypes within single or expanded TCR clonotypes. (**C**) Heatmap showing the similarity of TCRs among different samples. Similarity was measured with the Morisita index, and the similarity is indicated in the heatmap. (**D**) Bar plots showing the proportion of V gene usage in heavy (top) or light (bottom) chains. The proportion of gene usage was calculated separately in each sample. The color of the bar indicates the samples. (**E**) Dot plot showing the TCR diversity (measured by the inverse Simpson) in distinct samples. (**F**) UMAP plot showing the distribution of high-quality BCRs among all B cells and plasmablasts. (**G**) Boxplots showing the number of somatic hypermutations in heavy or light chains. Boxplot color indicates the donors. Wilcoxon test *P* values are indicated. *P < 0.05; **P < 0.01; ***P < 0.001; and ns, not significant.

After identifying differentially expressed genes (DEGs) before and after vaccination in COVID-19 convalescents and COVID-19 naive individuals, respectively, we further performed an overlap analysis between DEGs in these two populations (Fig. S8B and C). The highest number of DEGs that were specially upregulated in convalescents were detected in CD4^+^ naive T cells (290 DEGs), and these DEGs were not identified as upregulated DEGs in CD4^+^ naive T cells from COVID-19 naive individuals after vaccination (Fig. S8B). We found that these specially upregulated DEGs were involved in the adaptive immune system, mitogen-activated protein kinase family signaling cascades, class I major histocompatibility complex (MHC)-mediated antigen processing and presentation, and cellular protein catabolic process (Fig. 5D), which is related to cell activation and proliferation. Although vaccination resulted in the upregulation of genes related to the adaptive immune system and class I MHC-mediated antigen processing both in convalescents and COVID-19 naive individuals, the most striking upregulation of these genes occurred in CD4^+^ naive T cells from convalescents (~42.5% and 42.3% increase, respectively) (Fig. 5E). Finally, the analysis of TCR clonality showed that the single clonotypes were mainly from CD4^+^/CD8^+^ naive T cells and expanded clonotypes from CD4^+^/CD8^+^ T_EM_ cells and CD8^+^ T_TE_ cells (Fig. 6A and B). The TCR overlap analysis showed low similarity among different convalescents, reflecting the individual heterogeneity of the TCRs (Fig. 6C and D). We noticed that CD4^+^ T_EM_ cell clones expanded from 0.1% to 1.5% after vaccination, and the relative proportion of expanded clonotypes in CD8^+^ T_TE_ cells increased from 25.9% to 32.3%. Furthermore, T cell receptor (TCR) diversity increased in all three convalescents after vaccination (Fig. 6E), indicating potential T cell replenishment. The abundance of B-cell subtypes and plasmablasts exhibited no significant changes after vaccination (Fig. S7). B cell receptor (BCR) analysis was also performed. We detected that the number of somatic hypermutation in CDR3 was significantly increased only in a convalescent E01 (*P* = 0.002), which could be caused by analyzing all BCR sequences instead of antigen-specific BCRs (Fig. 6F and G).

Taken together, these results suggested that vaccination may induce the loss of activated T cells in COVID-19 convalescents, and therefore, plenty of naive T cells were generated to balance the immune environment.

## DISCUSSION

A live-attenuated prime inactivated booster vaccination strategy has been shown to induce an enhanced cellular response in addition to a specific humoral immune response (22). The scenario is similar to that of this study. Multiple studies have shown that immunization of previously infected individuals with one dose of an inactivated or mRNA vaccine results in increased anti-RBD and neutralizing antibody responses against the pseudotyped virus, surrogate virus, wild-type strain, and Delta variant, as well as elevated virus-specific B- and T-cell responses (23, 24). However, no studies were conducted to evaluate dynamic antibody changes in COVID-19 convalescents using live viral neutralization approaches.

Here, we conducted a multicenter prospective clinical cohort study on the earliest convalescents since the onset of the COVID-19 epidemic in China. We found that the neutralizing antibody titer against the live SARS-CoV-2 Omicron BA.5 variant in convalescents was reduced by 4.22-fold in 9–10 months. Therefore, we administered an inactivated vaccine dose to those convalescents with a neutralizing antibody titer lower than 1:96. We found that one booster dose resulted in a 4.84-fold increase in neutralizing antibodies against the live Omicron, validating the necessity and benefits of booster vaccination for COVID-19 convalescents with a waning neutralizing antibody after a long period of infection. The neutralizing antibody titer and the abundance of Ki67^+^ plasmablasts exhibited significant correlations, suggesting that the proliferation status of plasmablasts rather than the absolute number was more predictive of the specific antibody titer.

We observed that RBD-specific antibody levels increased, but neutralizing antibody titers decreased in convalescents after discharge and that vaccination induced an obvious increase in RBD-specific and neutralizing antibody titers. A previous study also found that although the titer of neutralizing antibodies in convalescents was low, they generally had a high level of RBD-specific antibodies (25). This indicates that although antibodies that could bind to RBD were continuously produced in convalescents after infection, the level of antibodies that could eliminate the virus was gradually reduced, and thus protection against SARS-CoV-2 infection waned, which deserves further investigation.

**Fig 7:**
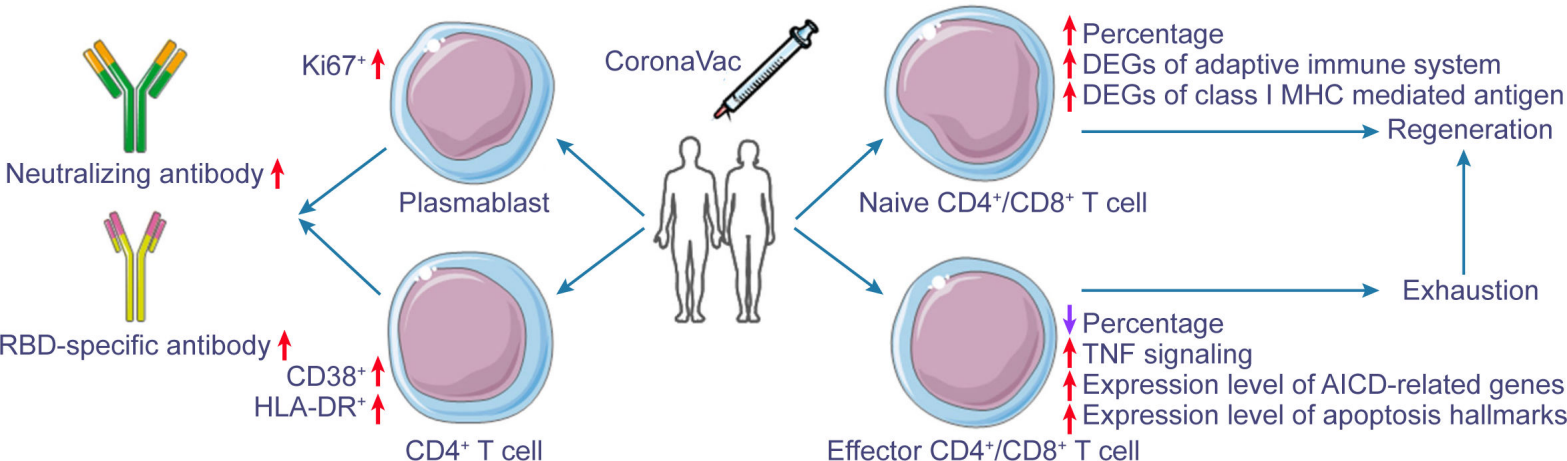
Dynamic changes in immune cells’ response pattern due to vaccination in COVID-19 convalescents. Regarding the humoral immune response, the expression of Ki67 in plasmablasts and the proportion of HLA-DR^+^CD38^+^ CD4^+^ T cells increased within 7 days after vaccination, further promoting the level of neutralizing antibodies and RBD-specific antibodies. In terms of cellular immune response, T cells showed an exhaustion and regeneration pattern after vaccination. Expression levels of TNF-mediated signaling pathways, AICD-related genes, and apoptosis-mediated signaling pathways increased in a short period. T cells were activated, the proportion of effector T cells gradually decreased, and T cells were gradually exhausted. At the same time, the proportion of naive T cells gradually increased, and plenty of naive T cells without antigen stimulation were supplemented to maintain the balance.

Interestingly, we also noticed that after immunization, the abundance of naive T cells increased significantly in convalescents; in contrast, the abundance of T_EMRA_ and T_EM_ cells decreased gradually, which was not observed in vaccinated COVID-19 naive individuals. Furthermore, non-naive CD4^+^ and CD8^+^ T cells exhibited transient or continuous activation after immunization (Fig. 3). Therefore, we hypothesized that certain antigen-experienced T cells were reactivated and gradually depleted after vaccination. As a result, the naive T cell population was replenished to maintain peripheral T-cell homeostasis (Fig. 7). The pattern of exhaustion and then replenishment of T cells upon vaccine stimulation and the upregulation of cytokine pathways in convalescents differed from the pattern of response in COVID-19 naive individuals after vaccination. COVID-19 infection affects the systemic immune environment, which may persist even after recovery, and inactivated vaccines may induce these pre-existing immune regulatory mechanisms. In fact, increased expression of activation and exhaustion markers in T cells was observed in convalescents, especially in patients with severe forms of the disease (22). Despite effective antibody enhancement, the exhaustion of the T cell response may be detrimental to the antiviral response, at least for a time. One study (26) also showed that the CD4^+^ T cell response in COVID-19 convalescents after enhanced immunization with vaccines exhibited a resting state, and no epitope-specific S1 T-cell response could be detected. Furthermore, the loss of correlation between the response of CD4^+^ T cells and the level of IgA antibody after booster immunization further indicates that CD4^+^ T cells in convalescents changed from active to resting state after booster immunization.

Our current study has several limitations. Although we conducted a multicenter, prospective clinical trial on COVID-19 convalescents, the size of our cohort is relatively small (*n* = 96). Considering the heterogeneity of convalescents and their complex immunological background, more people are needed, including those with different neutralizing antibody titers and immune responses to vaccines, to reach a more comprehensive conclusion. In addition, we assessed the neutralizing antibodies and T cell responses for 1 month after booster vaccination. Monitoring the long-term durability of neutralizing antibodies against the Omicron variant will be helpful. In fact, at this stage, we are still conducting continuous follow-up observations for all enrolled convalescents.

In conclusion, our study showed that neutralizing antibodies against Omicron decreased rapidly in a substantial proportion of COVID-19 convalescents. A booster dose in convalescents with low antibody levels could initiate a robust humoral response against the Omicron variant. Furthermore, vaccination also sensitized T cells to AICD and apoptosis, which was not observed in COVID-19 naive individuals. The immune mechanisms of vaccination that orchestrate protective antibodies and dampen T-cell responses and their impact on COVID-19 convalescents require further investigation.

## Data Availability

The processed 10× Genomics data supporting the findings of this study are available in Gene Expression Omnibus under the accession number GSE219086.

## SUPPLEMENTAL MATERIAL

Supplemental material spectrum.02435-23-s0001.docx Fig. S1 to S8, Tables S1 to S4, additional experimental details.

ASM does not own the copyrights to Supplemental Material that may be linked to, or accessed through, an article. The authors have granted ASM a non-exclusive, world-wide license to publish the Supplemental Material files. Please contact the corresponding author directly for reuse.
