# Supplementary material for: Increased antibody titers but induced T cell AICD and apoptosis response in COVID-19 convalescents by inactivated vaccine booster
Source: Microbiol Spectr. 2024 Feb 6;12(3):e02435-23. doi: 10.1128/spectrum.02435-23 (PMC10913726; doi:10.1128/spectrum.02435-23)
Supplement: Supplemental material — Fig. S1 to S8, Tables S1 to S4, additional experimental details. [file spectrum.02435-23-s0001.docx]

**Supporting Information**

**Peripheral blood mononuclear cells (PBMCs) isolation and flow cytometry**

It should be noted that among the 28 convalescents in the low titer neutralizing antibody group, only 20 convalescents had sufficient number of cell samples available for flow cytometry analysis at three indicated time. Similarly, among the 62 convalescents in the high titer neutralizing antibody group, only 17 convalescents had cell samples available for analysis. Venous blood (20-30 mL) from study volunteers was collected in EDTA tubes. The tubes were centrifuged at 400× g for 15 min to separate the cellular parts. The plasma-containing fraction was collected and centrifuged at 1200× g for 15 min, and the plasma was aliquoted and stored at -20°C. The cellular fraction was reconstituted with phosphate-buffered saline (PBS) and subjected to Ficoll density gradient centrifugation (500× g, 30 min) according to the manufacturer’s instructions. After washing, PBMCs were resuspended in 90% foetal bovine serum (FBS) and 10% dimethyl sulfoxide (DMSO, Sigma Life Science) and then frozen at -80°C for one night before being stored in liquid nitrogen. PBMCs were thawed at 37°C and washed twice before use.

Flow cytometry was used to detect the dynamic changes in T-cell and B-cell subsets after one inactivated vaccine booster dose. Approximately 1×10^6^ to 3×10^6^ isolated PBMCs were used per sample per stain. Supplementary Table S4 presents the antibody panel information. Briefly, PBMCs were stained with live/dead mix (100 µL, 30 min, RT) and washed with FACS buffer (1 × PBS with 2% FBS). Samples were then incubated with 100 µL of Fc block (RT, 15 min) before a second wash (FACS buffer, 1500 rpm, 5 min, RT). The pellet was resuspended in 50 µL of surface marker staining mix and incubated at RT for 30 min before acquiring data on an LSRII Fortessa (BD Bioscience). FCS files were analysed using FlowJo v10 software.

SARS-CoV-2-specific CD8^+^ T-cell responses were detected by tetramer staining. Briefly, two PE-labelled SARS-CoV-2-MHC tetramers (HLA-A2-S_269-277_ and HLA-A2-S_1000-1008_) were produced according to the manufacturer’s instructions for the QuickSwitchTM Quant HLA-A*02:01 Tetramer Kit (TB-7300-K1). PBMCs (2-4 × 10^6^) of HLA-A*02:01-positive samples were thawed, washed twice with PBS and stained with 5 µL HLA-A2-S_269-277_ or HLA-A2-S_1000-1008_ at room temperature for 1 h in FACS buffer. Samples were then incubated with FITC-labelled CD3 mAb and BV510-labelled CD8 mAb and stained with 7AAD for 5 minutes before acquiring data on an LSRII Fortessa (BD Bioscience). FCS files were analysed using FlowJo v10 software.

**Single-cell RNA-Seq**

Using single cell 5 library and Gel Bead Kit (10× Genomics, 1000006) and Chromium Single Cell A Chip Kit (10× Genomics, 120236), the cell suspension (300-600 living cells per microliter determined by Count Star) was loaded onto the Chromium single cell controller (10× Genomics) to generate single-cell gel beads in the emulsion according to the manufacturer's protocol. Briefly, single cells were suspended in PBS containing 0.04% BSA. Captured cells were lysed and the released RNA were barcoded through reverse transcription in individual GEMs. Reverse transcription was performed at 53℃ for 45 min on a S1000TM Touch Thermal Cycler (Bio Rad), followed by 85℃ for 5 min, and then hold at 4℃. The cDNA was generated and amplified, with quality assessment using an Agilent 4200 (performed by CapitalBio Technology, Beijing).

Using Single Cell 5’ Library and Gel Bead Kit, Single Cell V(D)J Enrichment Kit, Human T Cell (1000005) and Single Cell V(D)J Enrichment Kit, Human B Cell (1000016), single-cell RNA-seq libraries were constructed according to the manufacture’s instruction, respectively. Finally, the libraries were sequenced using an illumina Novaseg6000 sequencer with a sequencing depth of at least 100,000 reads per cell with pair-end 150 bp (PE150) reading strategy (performed by CapitalBio Technology, Beijing).

The procession of 10× gene expression data was performed using software *CellRanger*, in which process raw sequence data were mapped into the human reference genome (GRCh38). The obtained UMI matrix was processed to filter out genes detected in less than 10 cells and cells with less than 200 genes using the R package *Seurat* (version: 4.0.3) (27). Quality control was performed to obtain high-quality cells with the following two criteria: first, the number of detected genes greater than 400 and less than 6,000, and second, the proportion of transcripts mapping to mitochondrial genes less than 10%. The filtered cells were further processed to remove potential doublets using the Python package *scrublet* (28). Doublets were separately identified with the expected doublet rate of 6% for each sample, and cells with variable *doubletScores* greater than the 90% quantile were filtered out. Finally, 50,014 high-quantity cells were retained for the downstream analysis. An average of 4,062 UMIs and 1,567 genes were detected (Fig. S4B).

Gene expression levels were quantified with counts per million mapped reads (CPM) as the UMI number of a given gene in a given cell divided by the total UMI number of this given cell and then multiplied by 100,000. Gene expression levels were then transformed into log_2_(CPM + 1).

**Identifying main cell types**

The gene expression data quantified with raw UMI counts were used to identify cell types. To exclude the influence of batch effects, the scRNA-seq data of each sample were separately normalized using the function *NormalizeData* in the R package *Seurat* (parameter: *normilization.method* = *‘LogNormalize’, scale.factor* = *10000*), and we identified 3,000 highly variable genes for each sample using the function *FindVariableFeatues* with the ‘*vst*’ method. Then, 3,000 features were chosen for integrating multiple datasets using the function *SelectIntegrationFeatures*, and these selected features were utilized to find a set of anchors using the function *FindIntegrationAnchors*. Finally, we integrated scRNA-seq datasets from multiple samples with a set of anchors using the function *IntegrateData*. Principal component analysis was performed using the function *RunPCA*, and then components PC1-PC75 were selected to perform UMAP nonlinear dimensionality reduction analysis using the function *RunUMAP*. The unsupervised clustering analysis parameter *resolution* was set to three to separate the cells into fifty-three clusters. According to the expression level of well-known lineage marker genes, distinct clusters were merged, and eleven main cell types were determined.

The cluster of T cells (*n* = 23,785) was identified as one main cell type and was further separated into several cell subtypes. The gene expression data quantified with raw UMI counts of these T cells were used to identify cell subtypes. Multiple samples were integrated again with the same method using sample parameters as described for identifying the main cell types. We detected a cluster of platelets that highly expressed *PPBP* and a cluster of CD14^+^ monocytes that highly expressed *LYZ*, *FCN1* and *S100A8*. After removing these cells, we reintegrated the scRNA-seq datasets, leaving 23,536 cells, and separated these cells into twenty-one clusters with a clustering parameter *resolution* of one.

All these cells (*n* = 23,536) highly expressed marker genes of T cells, including *CD3D*, *CD3E*, *CD3G*, and *IL2RG*. We then identified a series of CD4 T cells based on marker genes, including CD4 naïve T cells (*CD4*, *IL7R*, and *CCR7*), CD4 memory T cells (*CD4* and *S100A4*) and CD4 effector memory T cells (*CD4*, *GZMK*, and *CCL5*). Cell subtypes of CD8 T cells were also revealed, including CD8 terminal effector T cells (*CD8A*, *GZMB*, and *HLA-DRB1*), CD8 effector memory T cells (*CD8A* and *GZMK*) and CD8 naïve T cells. We identified regulatory T (Treg) cells based on *RTKN2* and *FOXP3*, γδT cells based on *TRGV9* and *TRDV2*, and MAIT cells based on *TRAV1-2*.

We identified a main cluster of B cells and a cluster of plasmablasts (*n* = 3,835 in total) in the above analysis, and these cells were further separated into several cell subtypes. The gene expression data quantified with raw UMI counts of these cells were used to identify cell subtypes. Multiple samples were integrated again with the same method as described for identifying the main cells. We separated cells into ten clusters with a parameter *resolution* of one and detected a cluster of CD14^+^ monocytes. After removing these cells, 3,736 cells were left for downstream analysis. Then, scRNA-seq datasets were reintegrated and further separated into eight clusters with a parameter *resolution* of one. We identified plasmablasts based on the expression level of the marker gene *CD38*, naïve B cells based on *IGHD*, and memory B cells based on *CD27*.

DEGs before (day 0) and after vaccination (day 28) were separately calculated for each cell type or subtype. The log_2_-transformed gene expression data were loaded into the R package *Seurat*. A Wilcoxon rank sum test was applied to identify DEGs using the function *FindMarkers* with default parameters. The genes were chosen as DEGs only if the log_2_-transformed average difference was greater than 0.5, the percentage of expressed cells in the corresponding group (day 0 or day 28) was greater than 25%, the *P* value was less than 0.05, and the Benjamini & Hochberg false discovery rate (FDR) was less than 0.05. GO analysis was performed using the website tool *Metascape* with default parameters (29).

**Analysing T cell and B cell receptors**

The raw single-cell TCR sequencing data were processed with the software *CellRanger*, and then the filtered TCR contigs were obtained in files ‘*filtered_contigs.fasta*’ and ‘*filtered_contig_annotations.csv*’. Both TCRs and BCRs were detected in twenty-one cells that should be removed in TCR analysis and BCR analysis. Only TCRs in CD4 T cells, CD8 T cells and Treg cells identified based on scRNA-seq data were analysed. Then, TCRs were filtered out following three criteria: first, greater than two α-chain contigs and greater than two β-chain contigs; second, greater than two α-chain contigs or greater than two β-chain contigs, and third, only a single α-chain contig or a single β-chain contig. Therefore, only three types of TCRs were retained, including TCRs with one paired α-chain and β-chain contig, TCRs with two α-chain contigs and a single β-chain contig, and TCRs with a single α-chain contig and two β-chain contigs. Finally, high-quality TCRs were retained in 49.3% (9,042/18,338) of T cells (CD4 T cells, CD8 T cells and Treg cells).

Software *CellRanger* was used to assign the TCR clonotype for each single cell. TCR clonotypes were counted for each sample, and the clonotype corresponding to greater than two cells was considered an expanded clonotype and otherwise a single clonotype.

TCR overlap analysis was performed to measure the similarity between distinct samples. In this analysis, the Morisita index, an ecological measure of the dispersion of individuals within a population, was applied using the function *clonalOverlap* in the R package *scRepertoire* (version: 1.3.5) (30).

As described in the method of TCR analysis, filtered BCR contigs were generated after processing using software *CellRanger* and were stored in files ‘*filtered_contigs.fasta*’ and ‘*filtered_contig_annotations.csv*’. Both TCRs and BCRs were detected in twenty-one cells that should be filtered out, and only BCRs in B cells and plasmablasts identified based on scRNA-seq data were retained for further quality control assessment. Next, filtered BCR contigs were preprocessed before performing BCR analysis using the single-cell BCR-seq analysis package *dandelion* (version: 0.1.11), which was the Python package developed for 10× Chromium 5’ data in a previous study (31). Briefly, BCR contigs were first reannotated with function *reannotate_genes* using the IMGT reference database. Constant genes were then reannotated to correct assignment errors due to insufficient lengths of constant regions using function *assign_isotypes*.

Quality control assessment was then performed to retain high-quality BCRs using function *filter_contigs*. Briefly, BCRs matching the following four criteria were filtered out: first, contigs with mismatched loci and V, J and constant gene assignments; second, cell barcodes corresponding to multiple heavy-chain contigs; third, cell barcodes corresponding to multiple light-chain contigs, and fourth, cell barcodes corresponding to only light-chain contigs. Notably, BCRs matching criterion two were not removed if these BCRs matched three criteria: first, heavy-chain contigs were assessed to have identical V(D)J sequences, but software *CellRanger* assigned them as different contigs; second, contigs had a clear dominance of UMI counts, and third, one IgM and one IgD contig were assigned to a single-cell barcode. Finally, high-quality BCRs were retained in 74.7% (2,863/3,835) of B cells and plasmablasts.

Somatic hypermutations were calculated based on alignment sequences and corresponding germLine sequences using the function *observedMutations* in R package *shazam* (version: 1.1.0).

**Statistical analysis**

For comparison of the continuous variables, two-tailed Student’s *t*-test, repeated measures ANOVA, the Mann-Whitney U test, Wilcoxon-ranked *t*-test, and one-way ANOVA were performed and indicated in each figure legend. The one-tailed Pearson’s correlation analysis and the corresponding correlation test were performed and indicated in each figure length. The differences in categorical variables were evaluated using the chi-square and/or Fisher’s exact tests. Statistical analyses were conducted using *SAS* software (version 9.4; SAS Institute Inc., Cary, NC) or R language. The *P* values were indicated in each figure legend.


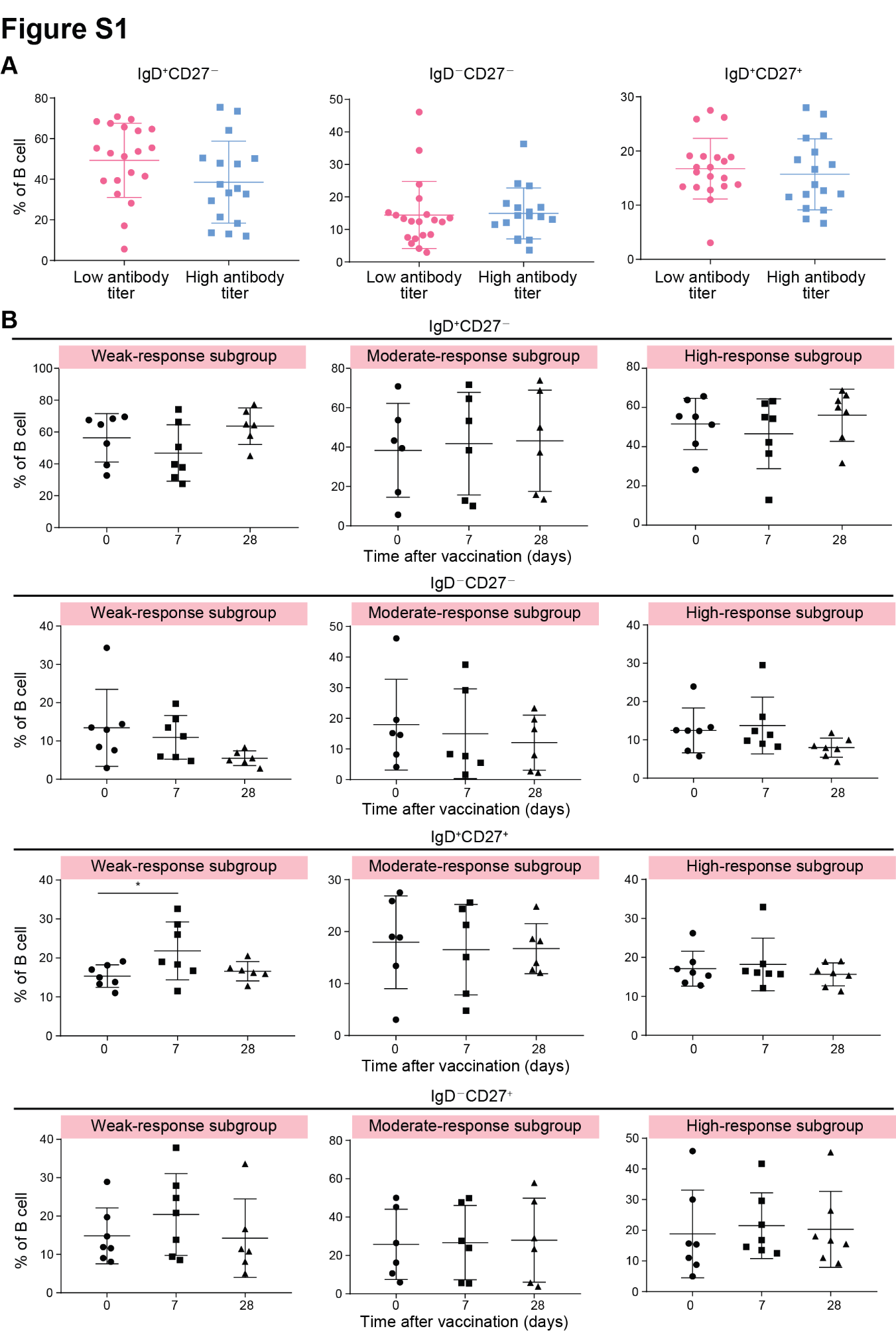


**Figure S1. Analysis of dynamic changes in B-cell subsets in COVID-19 convalescents.**

(A) Scatter plots showing the frequency of IgD^+^CD27^-^, IgD^-^CD27^-^, and IgD^+^CD27^+^ B cells in the low (*n* = 20) and high (*n* = 17) antibody titer groups. Data are shown as mean ± SD. A two-tailed Student’s *t*-test *P* value was computed.

(B) Scatter plots showing the frequency of IgD^+^CD27^-^, IgD^-^CD27^-^, IgD^+^CD27^+^, and IgD^-^CD27^+^ B cells in three vaccine subgroups of weak- (*n* = 7), moderate- (*n* = 6) and high-response (*n* = 7) to vaccination. Data are shown as mean ± SD. A two-tailed Student’s *t*-test *P* value was computed.


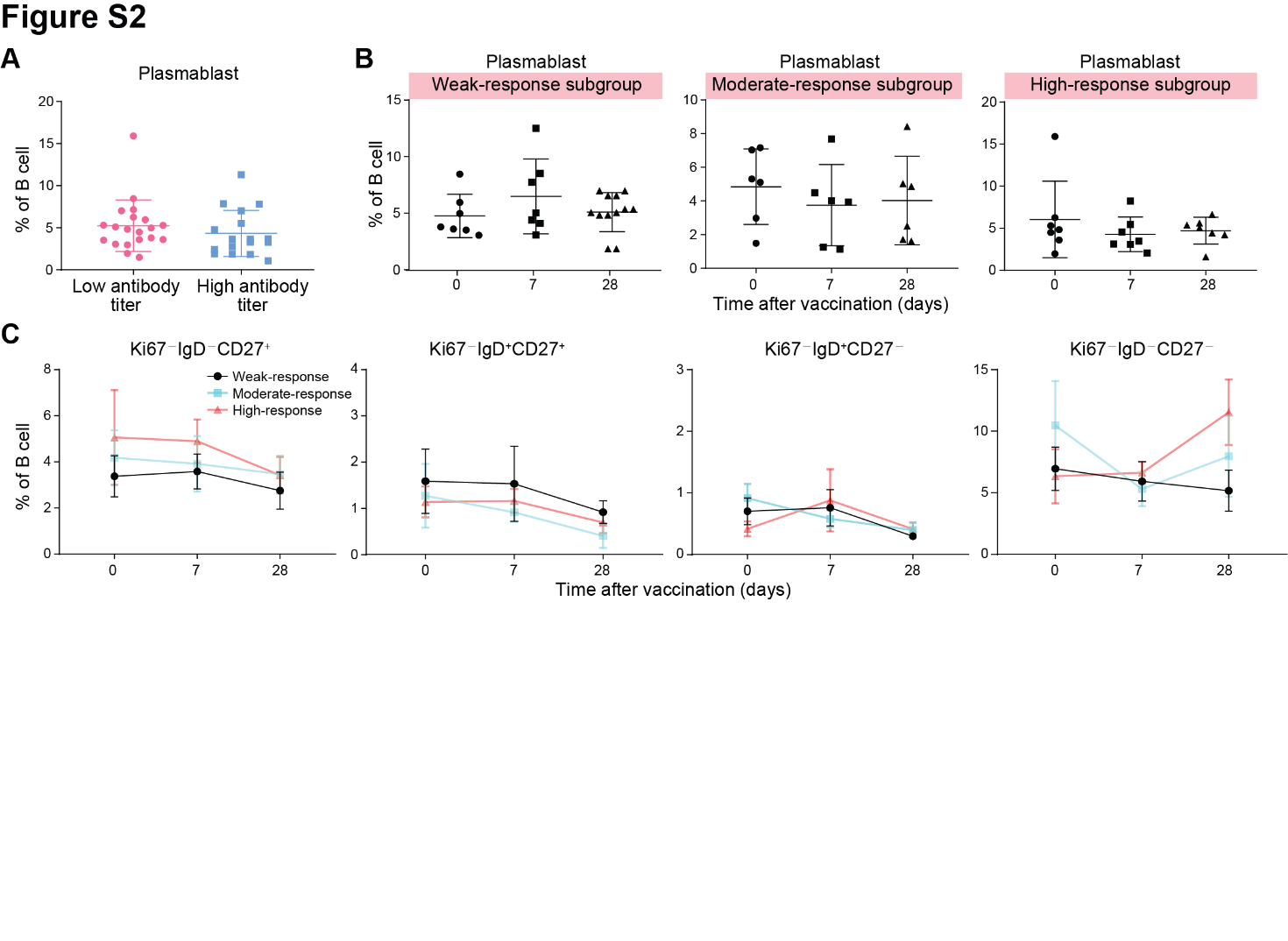


**Figure S2. Dynamic changes in plasmablasts and Ki67^+^ cells in four major B-cell subsets in COVID-19 convalescents.**

(A) Scatter plot showing the frequency of plasmablasts in the low and high antibody titer group. Data are shown as mean ± SD. A two-tailed Student’s *t*-test *P* value was computed.

(B) Scatter plot showing the frequency of plasmablasts in three vaccine subgroups of weak, moderate and high response to vaccination. Data are shown as mean ± SD. A two-tailed Student’s *t*-test *P* value was computed.

(C) Line plots showing the frequency of diverse B cell subsets in three vaccine subgroups of weak, moderate and high response to vaccination. Data are shown as mean ± SD. The statistical significance was calculated by one-way ANOVA.


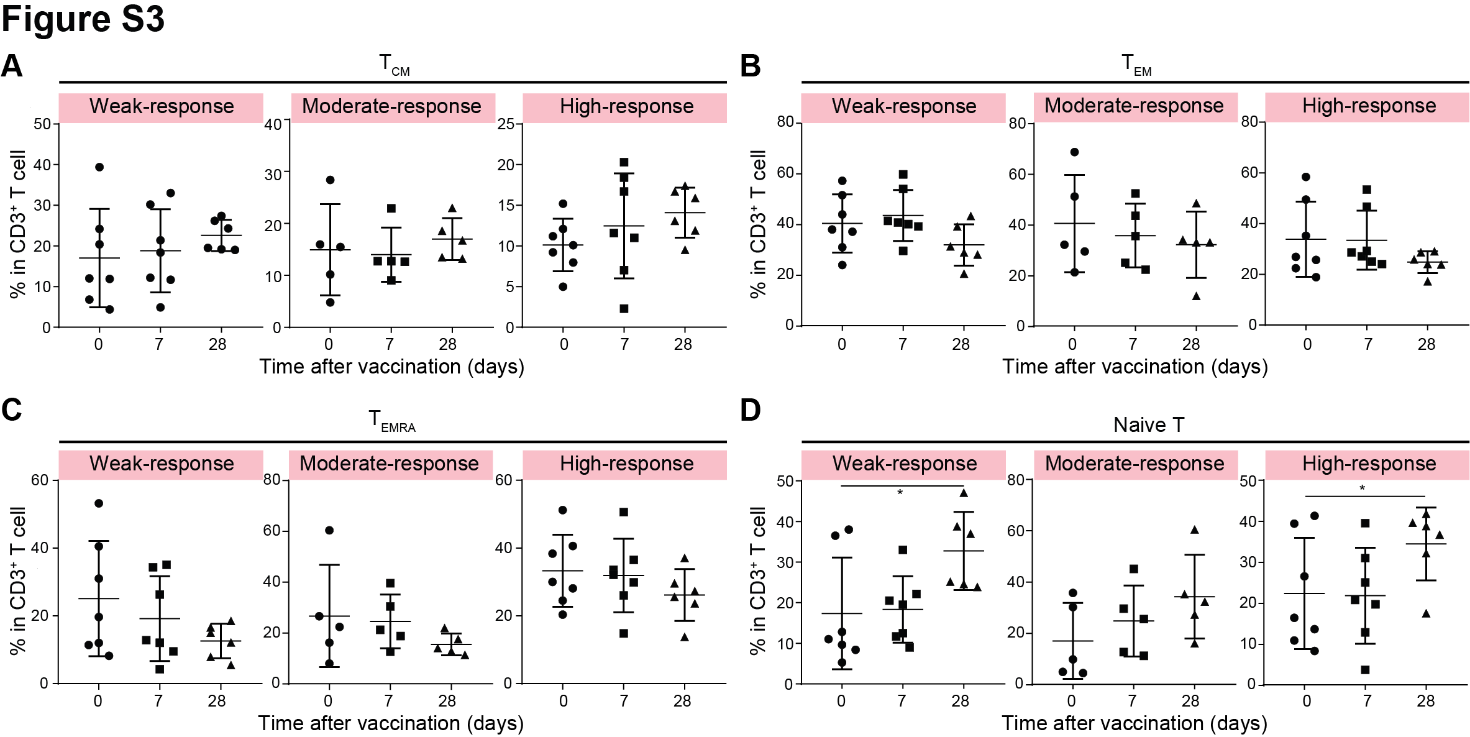


**Figure S3. Dynamic changes in the main subsets of T cells in the vaccine group.**

(A-D) Scatter plots showing the frequency of T_CM_ (A), T_EM_ (B), T_EMRA_ (C), naïve T (D) cell subsets in three subgroups of vaccines of weak, moderate, and high response to vaccination. Data are shown as mean ± SD. A two-tailed Student’s *t*-test *P* value was calculated.


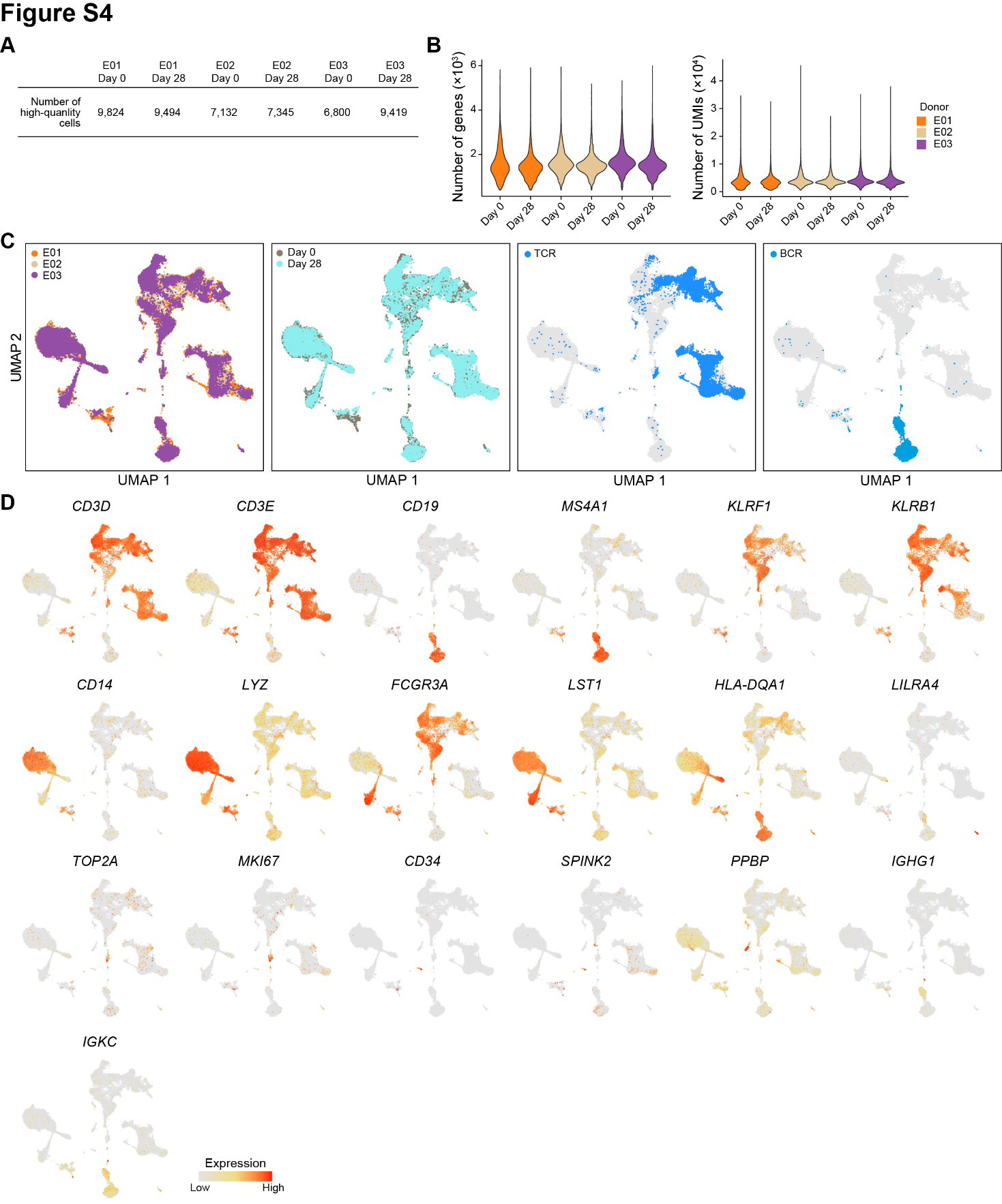


**Figure S4. Single-cell RNA sequencing reveals** **the immune landscape in COVID-19 convalescents before and after vaccination**

(A) Table showing the number of high-quality cells of three convalescents before and after vaccination.

(B) Violin plots showing the number of detected genes (left) and UMIs (right) in high-quality cells from each sample.

(C) UMAP plots showing donor and vaccination group information (left two panels) and detected TCR and BCR information (right two panels).

(D) UMAP plots showing the expression levels of well-known lineage marker genes.


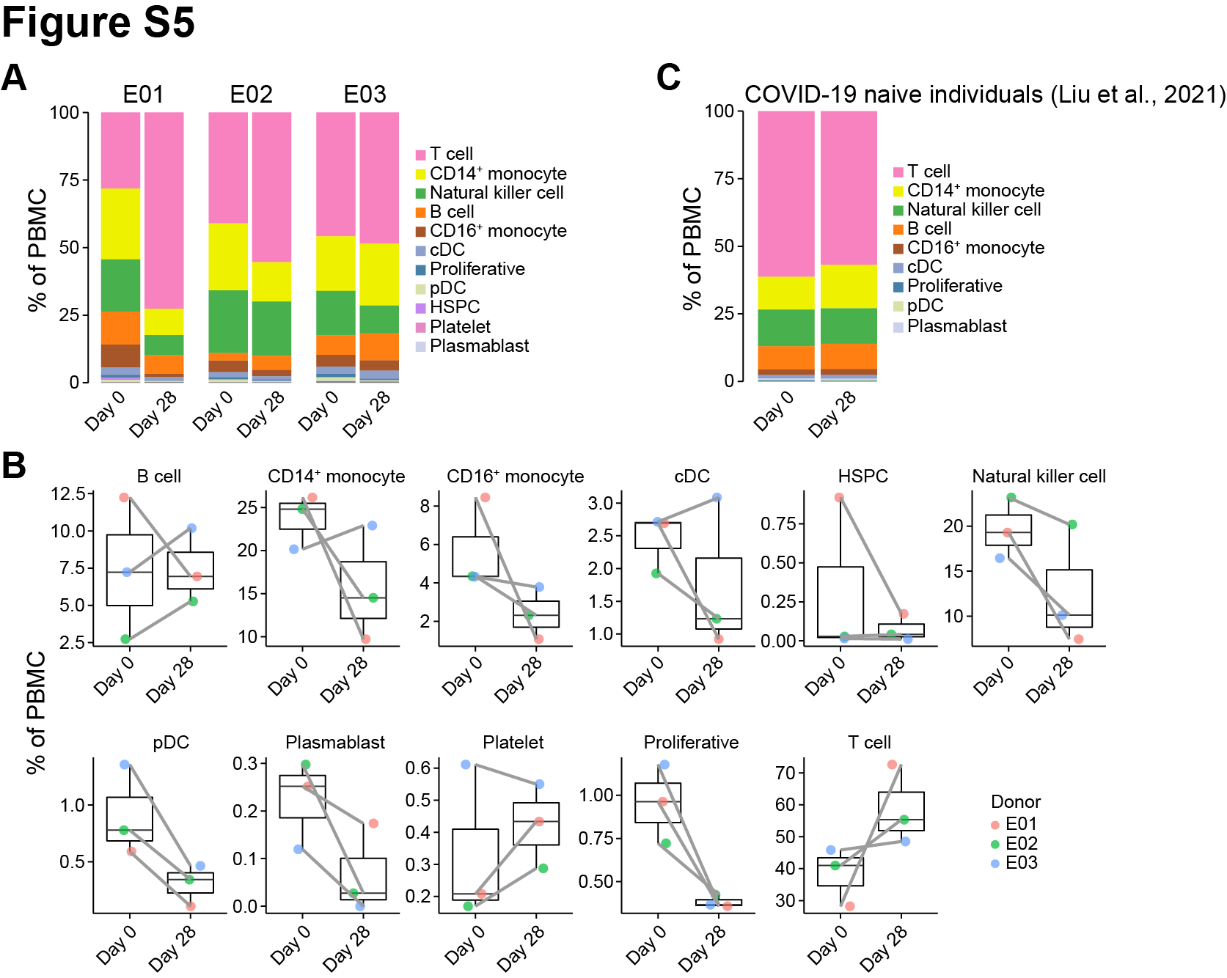


**Figure S5. Single-cell RNA sequencing reveals dynamic changes in immune cells after vaccination.**

(A) Stack bar plot showing the proportion of distinct cell types among all PBMC cells in each COVID-19 convalescent before and after vaccination.

(B) Boxplots showing the proportion of distinct cell types among all PBMCs. The color of the dot indicates the donor. Lines linked to the same donor.

(C) Stack bar graph showing the proportion of distinct cell types among all PBMC in COVID-19 naïve individuals before and after vaccination.


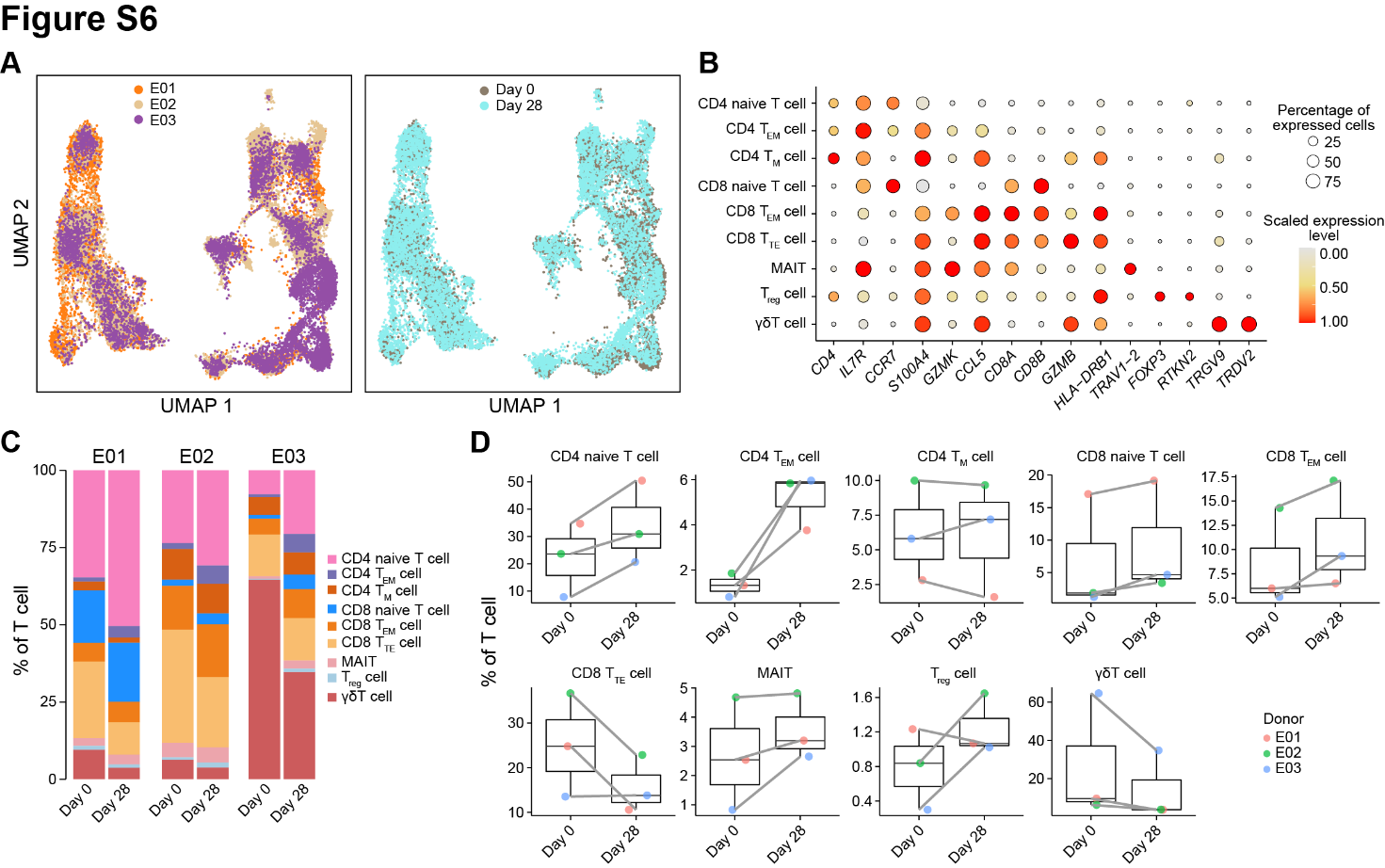


**Figure S6. Single-cell RNA sequencing reveals dynamic changes in T cells after vaccination.**

(A) UMAP plots showing the donor (left) and vaccination groups (right).

(B) Dot plot showing the expression levels of representative marker genes of T-cell subtypes. The size of the dot indicates the percentage of expressed cells, and the color of the dot indicates the scaled expression level. The expression level was scaled from zero to one among distinct cell subtypes.

(C) Stack bar graph showing the proportion of distinct T-cell subtypes among all T cells in each convalescent with COVID-19 before and after vaccination.

(D) Boxplots showing the proportion of distinct T-cell subtypes among all T cells. The color of the dot indicates the donor. Lines linked to the same donor.


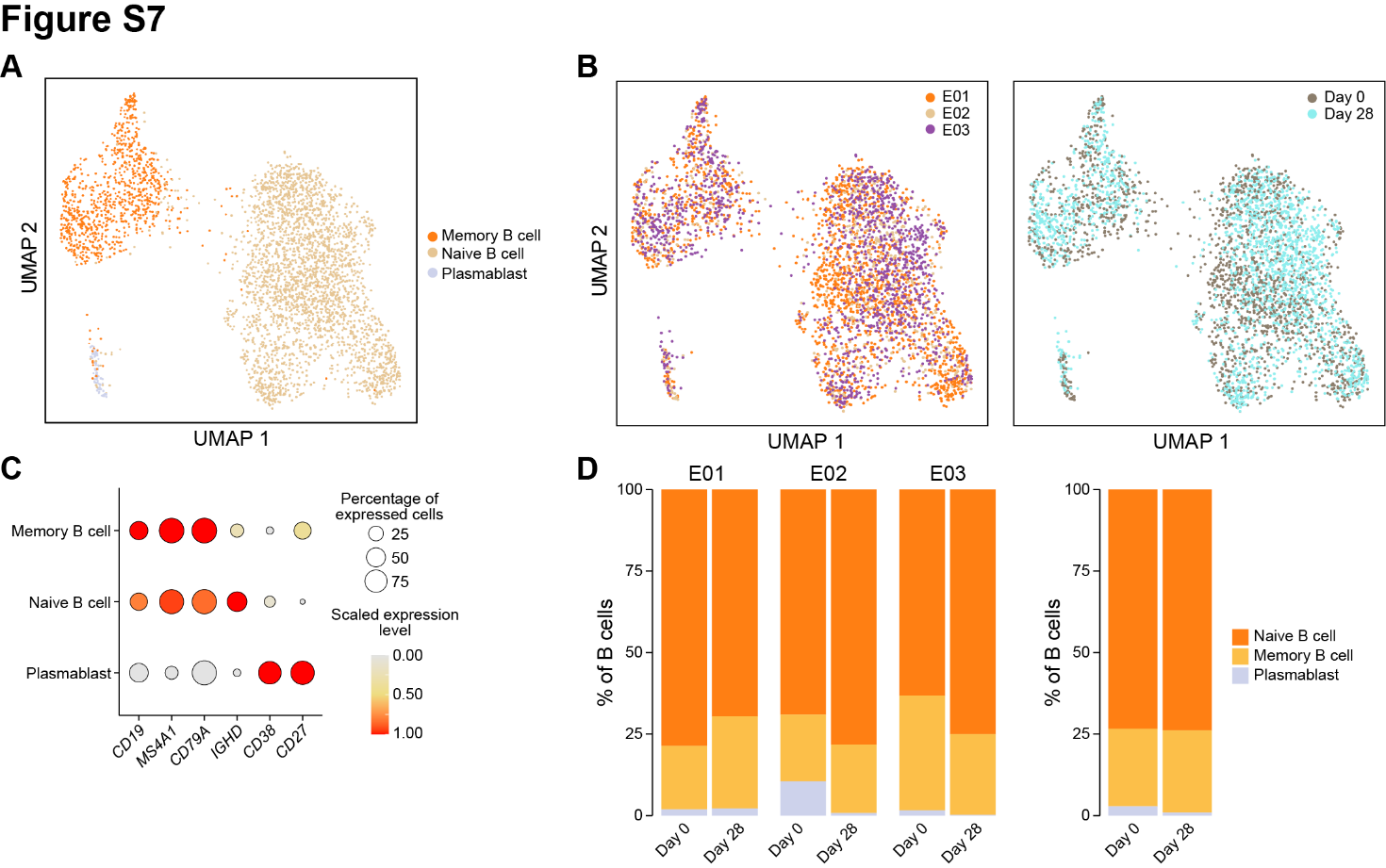


**Figure S7. Single-cell RNA sequencing reveals dynamic changes in B cells after vaccination.**

(A) UMAP plot showing B-cell subtypes and plasmablasts.

(B) UMAP plots showing the donor (left) and vaccination groups (right).

(C) Dot plot showing the expression levels of representative marker genes of B-cell subtypes and plasmablasts. The size of the dot indicates the percentage of expressed cells and the color of the dot indicates the scaled expression level. The expression level was scaled from zero to one among distinct cell subtypes.

(D) Stack bar graph showing the proportion of different B-cell subtypes and plasmablasts in each convalescent of COVID-19 before and after vaccination.


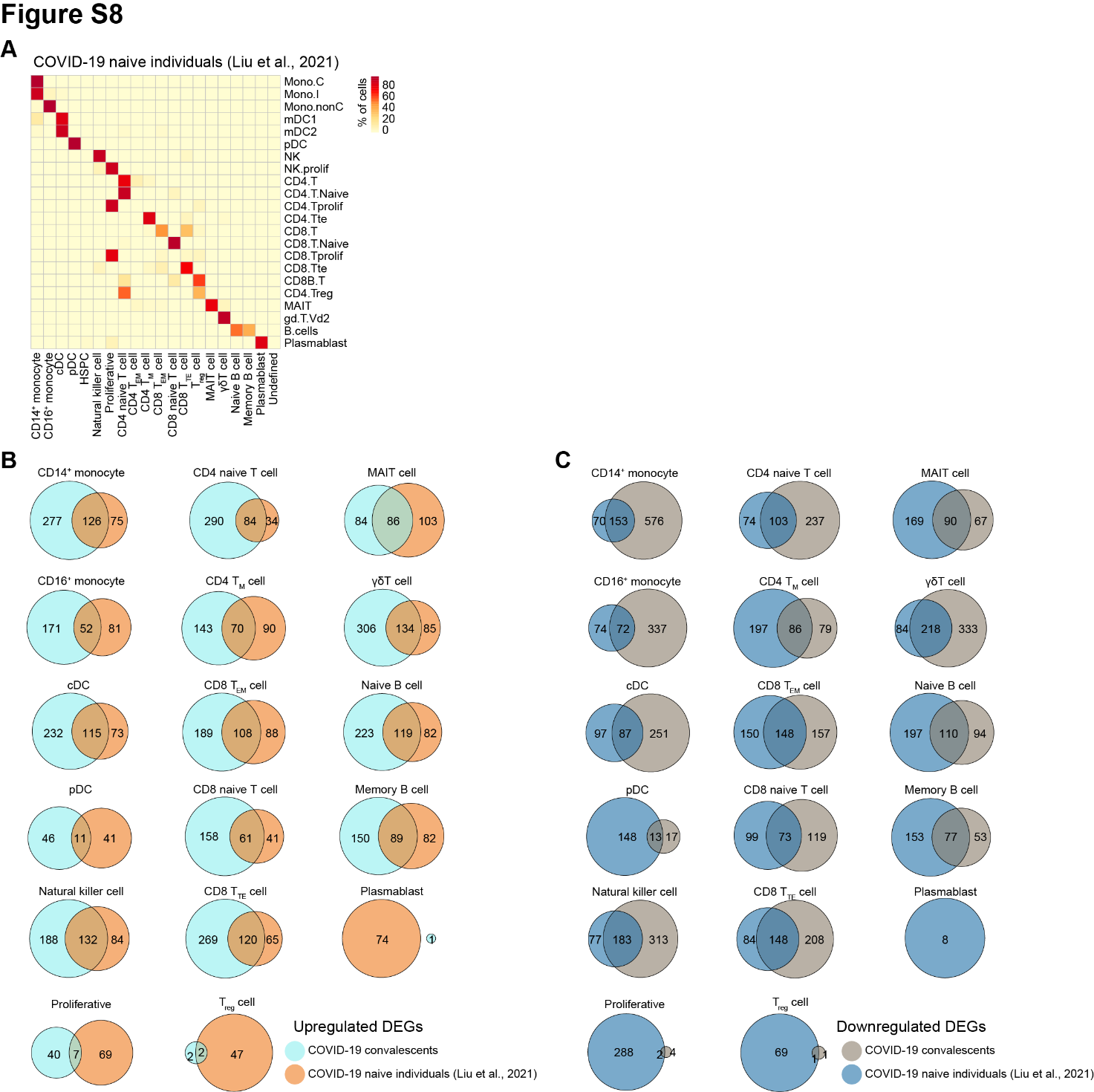


**Figure S8. Comparisons of transcriptomic changes due to vaccination in COVID-19 convalescents and COVID-19 naïve individuals.**

(A) Heatmap showing the proportion of cells assigned by two cell information sets in COVID-19 naïve individuals. The scRNA-seq data were obtained from a previous study. Bioinformatics analysis of this study obtained cell information in the column and cell information in the row was obtained directly from a previous study.

(B) Venn plots showing the result of the overlap upregulated DEGs in convalescents with COVID-19 and COVID-19 after vaccination. Cyan-blue and orange circles correspond to upregulated DEG in COVID-19 convalescents and COVID-19 naïve individuals, respectively. The number of overlapping DEGs, special DEGs of COVID-19 convalescents, or special DEGs of naïve individuals with COVID-19 is indicated.

(C) Venn plots showing the overlap result of DEGs downregulated in COVID-19 convalescents and COVID-19 naïve individuals after vaccination. The grey and blue circles correspond to DEGs downregulated in COVID-19 convalescents and COVID-19 naïve individuals, respectively. The number of DEGs that overlap, special DEGs of COVID-19 convalescents, or special DEGs of naïve individuals of COVID-19 is indicated.

**Supplementary Table 1. Baseline characteristics and laboratory findings of patients**

| Characteristics | Total  N = 90 | Severe patients  N = 24 | Non-severe patients  N = 66 | *P* |
| --- | --- | --- | --- | --- |
| Age (year) | 54 (46-61) | 57 (52-60) | 53 (43-62) | 0.097 |
| Sex (male) | 42 (46.67) | 13 (54.17) | 29 (43.94) | 0.390 |
| Exposure |  |  |  |  |
| Hubei province exposure | 8 (8.89) | 4 (16.67) | 4 (6.06) | 0.252 |
| Close contact with COVID-19 patient | 35 (38.89) | 12 (50.00) | 23 (34.85) | 0.192 |
| All of the above | 4 (4.44) | 2 (8.33) | 2 (3.03) | 0.288 |
| No exposure | 51 (56.66) | 10 (41.67) | 41 (62.12) | 0.083 |
| Coexisting medical conditions |  |  |  |  |
| Diabetes | 7 (7.78) | 2 (8.33) | 5 (7.58) | 1.000 |
| Hypertension | 18 (20.00) | 10 (41.67) | 8 (12.12) | 0.005 |
| Cardiovascular disease | 6 (6.67) | 1 (4.17) | 5 (7.58) | 1.000 |
| Endocrine system disease | 2 (2.22) | 1 (4.17) | 1 (1.52) | 0.464 |
| Respiratory system disease | 8 (8.89) | 3 (12.50) | 5 (7.58) | 0.759 |
| Signs and symptoms |  |  |  |  |
| Fever | 64 (71.11) | 20 (83.33) | 44 (66.67) | 0.123 |
| Cough | 50 (55.56) | 12 (50.00) | 38 (57.58) | 0.522 |
| Myalgia or fatigue | 34 (37.78) | 8 (33.33) | 26 (39.39) | 0.600 |
| Sputum production | 26 (28.89) | 8 (33.33) | 18 (27.27) | 0.575 |
| Headache | 9 (10.00) | 3 (12.50) | 6 (9.09) | 0.937 |
| Diarrhoea | 6 (6.67) | 4 (16.67) | 2 (3.03) | 0.041 |
| Dyspnoea | 14 (15.56) | 4 (16.67) | 10 (15.15) | 1.000 |
| Sore throat | 6 (6.67) | 2 (8.33) | 4 (6.06) | 0.656 |
| Rhinorrhoea | 3 (3.33) | 0 (-) | 3 (4.55) | 0.562 |
| Nausea and vomiting | 6 (6.67) | 3 (12.50) | 3 (4.55) | 0.336 |
| Chest pain and stuffiness | 8 (8.89) | 3 (12.50) | 5 (7.58) | 0.759 |
| Treatment |  |  |  |  |
| PEG-Interferon α2b | 70 (77.78) | 20 (83.33) | 50 (75.76) | 0.445 |
| Lopinavir/Ritonavir | 48 (53.33) | 9 (37.50) | 39 (59.09 | 0.069 |
| Antibiotic treatment | 35 (38.89) | 15 (62.50) | 20 (30.30) | 0.006 |
| Use of corticosteroid | 5 (5.56) | 5 (20.83) | 0 (-) | 0.001 |
| Intravenous immunoglobulin therapy | 6 (6.67) | 4 (16.67) | 2 (3.03) | 0.041 |
| Chinese traditional medicine | 56 (62.22) | 14 (58.33) | 42 (63.64) | 0.646 |
| Long COVID-19 | 11 (12.22) | 2 (8.33) | 9 (13.64) | 0.753 |
| Biochemistry |  |  |  |  |
| TP 60-83(g/L) | 68.79 (64.35-71.85) | 66.50 (61.51- 70.29) | 69.15 (65.79-72.31) | 0.011 |
| Albumin 35-55(g/L) | 38.00 (34.50-40.40) | 36.45 (33.50- 38.60) | 38.90 (35.90-40.80) | 0.005 |
| Globumin 20-40(g/L) | 30.46 (26.80-33.83) | 30.37 (27.19- 33.24) | 30.56 (26.70-34.80) | 0.719 |
| A/G 1.2-2.4 | 1.26 (1.08-1.49) | 1.25 (1.06-1.41) | 1.26 (1.10-1.50) | 0.286 |
| DBiL 0-6.8 (μmol/L) | 3.15 (1.90-5.60) | 3.30 (2.42- 5.51) | 3.05 (1.87-5.60) | 0.522 |
| TBiL 3.4-20.5 (μmol/L) | 9.72 (6.90-13.50) | 10.34 (6.61- 15.55) | 9.66 (7.19-13.30) | 0.809 |
| D/T | 0.37 (0.25-0.48) | 0.42 (0.28-0.50) | 0.35 (0.23-0.46) | 0.355 |
| ALT 5-40 (U/L) | 23.85 (15.00-38.30) | 27.40 (17.40 -37.50) | 22.20 (14.60-38.30) | 0.535 |
| AST 5-40 (U/L) | 31.50 (22.00-41.90) | 32.30 (26.50-46.95) | 29.10 (21.50-41.00) | 0.272 |
| S/T | 0.94 (0.70-1.45) | 0.94 (0.74-1.47) | 0.94 (0.69-1.45) | 0.925 |
| ALP 40-150 (U/L) | 54.00 (40.00-69.00) | 49.50 (39.00-61.50) | 55.00 (41.00-69.00) | 0.613 |
| GGT 11-50 (U/L) | 26.00 (18.00-45.00) | 36.50 (19.50-66.00) | 25.50 (18.00-44.00) | 0.165 |
| Cholinesterase 5000-12000 (U/L) | 6532 (5872-7548) | 6010 (4672-6637) | 6704 (6123-7787) | 0.002 |
| Urea 2.9-8.2 (mmol/L) | 4.05 (3.30-4.69) | 3.70 (3.43-5.34) | 4.07 (3.19-4.67) | 0.813 |
| Creatinine 62-115 (μmol/L) | 58.70 (50.00-70.00) | 64.00 (54.50-70.50) | 57.00 (50.00-68.00) | 0.463 |
| UA 208-428 (μmol/L) | 219.50 (179.00-264.00) | 216 (178-265) | 223 (193-264) | 0.544 |
| Glucose 3.9-6.1 (mmol/L) | 5.95 (5.21-7.20) | 7.05 (5.51-8.91) | 5.85 (5.11-6.40) | 0.009 |
| TC 2.8-5.2 (mmol/L) | 3.70 (3.35-4.15) | 3.76 (3.23-4.06) | 3.68 (3.36-4.20) | 0.456 |
| Triglyceride 0.56-1.7 (mmol/L) | 1.13 (0.88-1.61) | 1.08 (0.85-1.28) | 1.19 (0.89-1.67) | 0.225 |
| HDL-c 1.29-1.55 (mmol/L) | 1.06 (0.85-1.21) | 0.94 (0.81-1.17) | 1.07 (0.93-1.23) | 0.114 |
| LDL-c 2.1-3.1 (mmol/L) | 2.14 (1.80-2.55) | 2.26 (1.69-2.61) | 2.11 (1.84-2.53) | 0.923 |
| Apo-A1 1.05-2.05 (g/L) | 0.92 (0.82-1.07) | 0.80 (0.69-0.95) | 0.98 (0.86-1.07) | < 0.001 |
| Apo-B 0.55-1.30 (g/L) | 0.79 (0.65-0.95) | 0.80 (0.69-0.94) | 0.75 (0.65-0.95) | 0.861 |
| ADA 0-20 (U/L) | 21.15 (16.75-23.95) | 21.95 (18.90-25.10) | 20.00 (16.50-23.10) | 0.152 |
| Calcium 2.03-2.54 (mmol/L) | 2.16 (2.10-2.24) | 2.12 (2.06-2.20) | 2.17 (2.11-2.25) | 0.025 |
| Phosphorus 0.97-1.45 (mmol/L) | 0.97 (0.87-1.10) | 0.91 (0.79-1.01) | 1.00 (0.89-1.16) | 0.004 |
| Magnesium < 1.25 (mmol/L) | 0.92 (0.84-1.02) | 0.96 (0.80-1.02) | 0.91 (0.84-1.02) | 0.932 |
| CK 18.0-198.0 (U/L) | 77 (49-165) | 115 (51-209) | 76 (47-141) | 0.301 |
| CKMB < 0.6 ng/ml | 7.55 (5.00-11.00) | 8.75 (6.30-15.00) | 6.80 (4.20-9.70) | 0.068 |
| NT-proBNP 0-125 (pg/ml) | 95 (42-224) | 119 (45-301) | 66 (42-171) | 0.355 |
| LDH 109-245 (U/L) | 278 (227-340) | 340 (301-414) | 264 (210-309) | < 0.001 |
| CRP 0.068-8.2 (mg/L) | 27.72 (12.08-77.63) | 48.55 (26.70-85.50) | 20.57 (7.20-46.90) | 0.004 |
| ESR 0-15 (mm/60min) | 46.85 (27.00-76.80) | 33.60 (20.00-81.90) | 47.70 (32.20-75.00) | 0.564 |
| Blood routine |  |  |  |  |
| WBC 3.97-9.15 (10^9^/L) | 5.28 (4.26-6.99) | 5.18 (3.96-7.84) | 5.28 (4.36-6.83) | 0.993 |
| NEUT 2-7 (10^9^/L) | 3.62 (2.49-4.47) | 4.06 (2.55-5.90) | 3.56 (2.41-4.30) | 0.206 |
| LYMPH 0.8-4.0 (10^9^/L) | 1.24 (0.88-1.69) | 0.89 (0.60-1.21) | 1.39 (1.02-1.83) | < 0.001 |
| MONO 0.12-1.0 (10^9^/L) | 0.37 (0.25-0.57) | 0.28 (0.15-0.49) | 0.44 (0.27-0.58) | 0.020 |
| EO 0.02-0.5 (10^9^/L) | 0.02 (0.01-0.08) | 0.01 (0.00-0.03) | 0.04 (0.01-0.10) | 0.012 |
| BASO 0-1 (10^9^/L) | 0.01 (0.01-0.02) | 0.01 (0.00-0.02) | 0.02 (0.01-0.02) | 0.006 |
| RBC 4.09-5.74 (10^12^/L) | 4.16 (3.85-4.51) | 4.34 (3.91-4.57) | 4.13 (3.84-4.50) | 0.312 |
| Haemoglobin 131-172 (g/L) | 131 (118-140) | 133 (123-143) | 130 (117-139) | 0.344 |
| PCV 38-50.8 (%) | 39.00 (35.65-42.15) | 39.70 (36.05-42.10) | 38.75 (35.50-42.30) | 0.822 |
| MCV 83.9-99.1 (fL) | 93.15 (90.25-96.25) | 91.25 (88.30-95.80) | 93.80 (91.30-96.25) | 0.072 |
| MCHC 27.8-33.8 (pg) | 31.00 (29.90-31.95) | 31.20 (29.35-32.15) | 30.95 (30.05-31.85) | 0.764 |
| RDW 35.0-56.0 (fl) | 42.60 (40.00-44.70) | 42.35 (39.70-43.05) | 42.80 (41.00-44.80) | 0.176 |
| PLT 85-303 (10^9^/L) | 160 (129-204) | 147 (118-187) | 166 (134-206) | 0.314 |
| PCT 0.06-0.40 (%) | 0.02 (0.02-0.15) | 0.06 (0.02-0.16) | 0.02 (0.02-0.15) | 0.430 |
| MPV 7.54-11.24 (fL) | 9.30 (8.85-10.35) | 9.60 (9.20-10.65) | 9.20 (8.75-10.00) | 0.073 |
| PDW 9.0-18.0 (%) | 16.10 (15.70-16.30) | 16.20 (15.70-16.50) | 16.00 (15.70-16.30) | 0.166 |
| Coagulation routine |  |  |  |  |
| PT 11-13 (s) | 12.05 (11.40-12.80) | 12.15 (11.50-12.70) | 11.90 (11.10-12.80) | 0.503 |
| INR 0.8-1.2 | 1.05 (1.00-1.11) | 1.08 (1.00-1.15) | 1.04 (1.00-1.10) | 0.234 |
| D-dimer < 0.55 (mg/L) | 0.56 (0.31-0.86) | 1.08 (0.56-1.58) | 0.47 (0.28-0.69) | < 0.001 |

Abbreviations: TP, total protein; DBiL, direct bilirubin; TBiL, total bilirubin; ALT, alanine aminotransferase; AST, aspartate aminotransferase; ALP, alkaline phosphatase; GGT, γ-glutamyl transferase; UA, uric acid; TC, total cholesterol; HDL-c, high density lipoprotein cholesferol; LDL-c, low density lipoprotein cholesterol; Apo A1, apolipoprotein A1; Apo B, apolipoprotein B; ADA, adenosine deaminase; CK, creatine kinase; BNP, brain natriuretic peptide; LDH, lactate dehydrogenase; CRP, C-reactive protein; ESR, erythrocyte sedimentation rate; WBC, white blood cell; NEUT, absolute neutrophil count; LYMPH, absolute lymphocyte value; MONO, absolute monocyte; EO, absolute eosinophil; BASO, absolute basophilic; RBC, red blood cell; PCV, packed cell volume; MCV, mean corpuscular volume; MCHC, mean corpuscular hemoglobin concentration; RDW, red blood cell distribution width; PLT, platelet count; PCT, platelet hematocrit; MPV, mean platelet volume; PDW, platelet distribution width; PT, prothrombin time; INR, international normalized ratio. Data and samples of severe patients were collected on the day when they were diagnosed to be severe. Data and samples of non-severe patients were collected in admission.

**Supplementary Table 2. The follow-up of COVID-19 convalescents (beyond 12 weeks from initial symptoms)**

| **Patients** | **Gender** | | **Age (year)** | **Symptoms** | | | | | | | |
| --- | --- | --- | --- | --- | --- | --- | --- | --- | --- | --- | --- |
| N=11 | Female | Male | 50 (40-60) | Alopecia | Chest distress | Fatigue | Insomnia | Rhinitis | Olfactory loss (early phase) | Hypoimmunity | Osteodynia |
| 11/90  (12.22%) | 8/11  (72.73%) | 3/11  (27.27%) |  | 3/90  (3.33%) | 2/90  (2.22%) | 3/90  (3.33%) | 2/90  (2.22%) | 1/90  (1.11%) | 1/90  (1.11%) | 1/90  (1.11%) | 1/90  (1.11%) |

**Supplementary Table 3. Adverse events (during 0-7 days after vaccination)**

| **Patients** | **Gender** | | **Age (year)** | **Symptoms** | |
| --- | --- | --- | --- | --- | --- |
| N=3 | Female | Male | 58 (33-60) | Transient headache | Transient BT elevation |
| 3/28 (10.71%) | 1/28 (3.57%) | 2/28 (7.14%) |  | 1/28 (3.57%) | 2/28 (7.14%) |

**Supplementary Table 4. Flow cytometry antibody information**

| **Vendor** | **Antibody** | **Dye** | **Clone** | **Cat.#** |
| --- | --- | --- | --- | --- |
| Biolegend | CD3 | FITC | OKT3 | 317305 |
| Biolegend | CD4 | PerCP-cy 5.5 | OKT4 | 317427 |
| Biolegend | CD8 | BV510 | SK1 | 344732 |
| Biolegend | CD27 | APC-cy7 | O323 | 302815 |
| Biolegend | CD45RA | PE-CF594 | HI100 | 304145 |
| Biolegend | CCR7 | BV650 | G043H7 | 353233 |
| Biolegend | Ki-67 | BV421 | Ki-67 | 350505 |
| Biolegend | CD38 | PE | HIT2 | 303505 |
| Biolegend | HLA-DR | BV605 | L243 | 307639 |
| Biolegend | CD19 | BV510 | HIB19 | 302241 |
| Biolegend | CD38 | BV605 | HIT2 | 303531 |
